# Polyphenolic metabolites in *Scutellaria baicalensis* as potential candidate agents for the treatment of ischemic stroke

**DOI:** 10.3389/fphar.2025.1700164

**Published:** 2025-11-13

**Authors:** Jinlong Zhang, Songzhe Li, Lina Huang, Xicheng Jiang

**Affiliations:** College of Basic Medicine, Heilongjiang University of Chinese Medicine, Harbin, China

**Keywords:** polyphenol, ischemic stroke, *Scutellaria baicalensis*, baicalein, neurological deficit, traditional Chinese medicine

## Abstract

**Objective:**

This study aims to elucidate the therapeutic effects of polyphenolic metabolites from *Scutellaria baicalensis* Georgi against ischemic stroke. The findings are expected to provide experimental evidence and novel insights to guide the future development of these metabolites.

**Materials and methods:**

This review was conducted based on a comprehensive literature search of the PubMed, NCBI, and Google Scholar databases from their inception until August 2025. Key search terms included “Scutellaria baicalensis”, “Scutellaria baicalensis and polyphenols,” “Ischemic stroke,” “cerebral infarction,” “cerebral ischemia-reperfusion injury,” and “toxicity.” The article first summarizes the polyphenolic metabolites of S. baicalensis, such as baicalein, baicalin, wogonin, wogonoside, scutellarin, chrysin, apigenin, chlorogenic acid, and ferulic acid, and provides an overview of the pathophysiological mechanisms of ischemic stroke. The primary focus lies on elucidating the pharmacological mechanisms, potential toxic effects, and strategies for improving the bioavailability of these polyphenols in the treatment of ischemic stroke.

**Results:**

The polyphenolic metabolites of *S. baicalensis* significantly alleviate ischemic brain injury through multiple pharmacological mechanisms, including anti-inflammatory, antioxidant, and anti-apoptotic effects, as well as regulation of neurotransmitters, maintenance of the blood-brain barrier, and inhibition of ferroptosis, thereby demonstrating promising neuroprotective potential. Furthermore, although nanodelivery systems can effectively enhance the brain bioavailability of these metabolites, their dose-dependent toxicity requires careful attention.

**Conclusion:**

The polyphenolic metabolites of *S. baicalensis* exhibit promising development prospects due to their synergistic therapeutic effects on ischemic stroke via multi-targets and multi-pathways. To advance these metabolites toward clinical application, a strategic focus on the optimization of delivery systems and comprehensive safety assessment is imperative.

## 1 Introduction

Stroke is a cerebrovascular disease characterized by sudden vascular impairment that leads to neurological deficits (Zhang C. et al., 2025). Due to its severity, unpredictability, and uncontrollable nature, stroke is known for its high rates of disability and mortality, ranking as the second leading cause of death and the third leading cause of disability worldwide (Gan et al., 2025; He et al., 2024). Clinical studies indicate that stroke patients often develop various sequelae, including neurological deficits, hemiplegia, anxiety, and depression (He et al., 2024). Projections suggest that global stroke-related mortality is expected to rise by 50% between 2020 and 2050, increasing from 6.6 million to 9.7 million annual deaths (Feigin et al., 2023). Notably, the global economic burden of stroke exceeds $890 billion USD, with this impact being particularly pronounced in low- and middle-income countries (Feigin et al., 2025). Thus, stroke imposes a substantial burden and significant economic pressure on patients, their families, and society as a whole.

Pathologically, stroke can be classified into two subtypes: hemorrhagic stroke and ischemic stroke (Peng et al., 2025). Ischemic stroke accounts for the majority of all stroke cases (approximately 80%) (Irisa and Shichita, 2025; Liu Y. et al., 2025). Its pathological mechanism primarily involves cerebral vascular occlusion or stenosis, leading to reduced local cerebral blood perfusion, which subsequently triggers ischemic-hypoxic necrosis and neurological deficits (Li D. et al., 2025). Currently, intravenous thrombolysis and endovascular thrombectomy are established as primary treatment strategies for ischemic stroke and can effectively reduce the risk of disability (Yu M. et al., 2025). However, the narrow therapeutic window of these treatments (0–4.5 h after onset (Hernandez et al., 2025)), means that many patients arrive too late to benefit from them. Moreover, even after successful vascular recanalization and restoration of blood flow, further damage to the tissue and microcirculation may occur, resulting in cerebral ischemia-reperfusion injury (Yu Y. et al., 2025). In terms of drug development, several single-target therapeutic agents, such as natalizumab and nerinetide (NA-1), face limitations in clinical applicability or offer only modest improvements in outcomes (Paul and Candelario-Jalil, 2021). Therefore, there is an urgent need to explore multi-target synergistic intervention strategies. Developing safer and more effective therapeutic approaches for ischemic stroke remains a critical focus of current research.

Chinese herbal medicines (CHMs) contain a variety of bioactive metabolites and exhibit multi-target characteristics (Pang et al., 2024), offering unique advantages in the treatment of complex diseases. *Scutellaria baicalensis* (SB), the dried root of *S. baicalensis* Georgi (Lamiaceae), is widely distributed in northern, northwestern, and southwestern China, as well as in Japan, Korea, Russia, Mongolia, and other regions of South Asia (Yang R. et al., 2024; Arumugam et al., 2025). In traditional Chinese medicine, SB is typically harvested in spring and autumn, dried, and used directly as an botanical drug (Dzięcioł et al., 2024), it is known for its functions in “clearing heat and dampness, detoxifying, stopping bleeding, relieving diarrhea, and calming the fetus” (Zhao et al., 2024). Notably, Xiaoxuming Decoction, a classical formula from *Essential Prescriptions for Emergencies* used to treat stroke, relies significantly on the role of SB. Chemical analysis revealed that metabolites derived from SB account for 21% of the total metabolites in this formula, suggesting its substantial contribution to the therapeutic effects against ischemic stroke (Luo et al., 2019). Modern pharmacological studies have shown that polyphenolic metabolites from SB, including flavonoids and phenolic acids, can effectively mitigate ischemic stroke and prevent post-ischemic neurodegenerative damage (Zhao et al., 2019; Lu et al., 2011; Duda-Chodak and Tarko, 2023; Lin, 2011). These effects are mediated through multiple mechanisms such as antioxidant, anti-apoptotic, and anti-inflammatory activities (Liang et al., 2017), reflecting a broad spectrum of biological interventions.

This review focuses on the pharmacological effects of polyphenolic metabolites derived from SB in the treatment of ischemic stroke.

## 2 Polyphenolic metabolites in *Scutellaria baicalensis*

**FIGURE 1:**
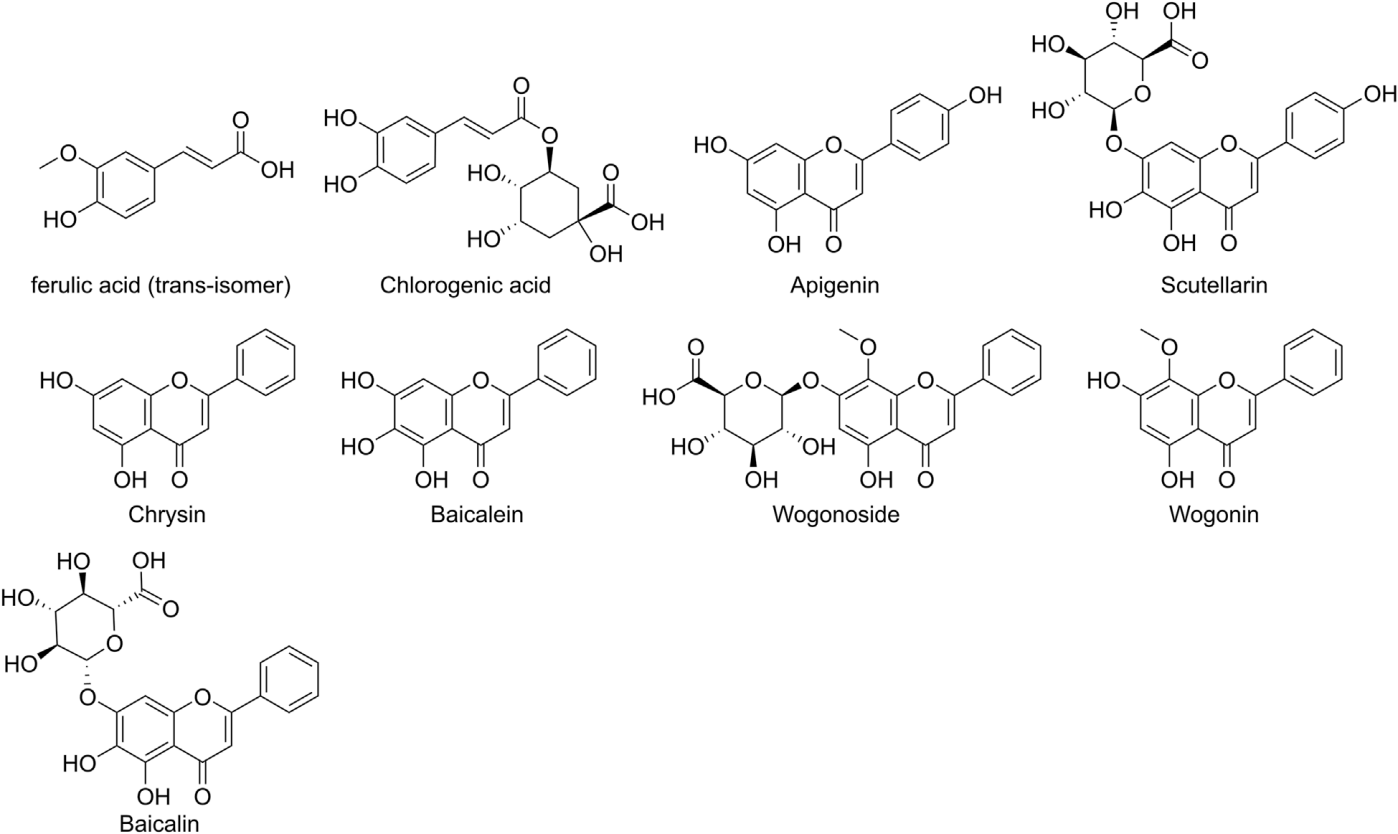
Chemical Structures of Major Polyphenolic Metabolites in *Scutellaria baicalensis*. Note: Ferulic acid is depicted as the trans-isomer, as this is the predominant form in nature and corresponds to the isomer used in the studies reviewed herein.

SB is one of the most important botanical drug sources of polyphenols (Jalili et al., 2024). Studies have identified multiple polyphenolic metabolites in its extracts, including baicalein (Rahmani et al., 2022), baicalin (Takahashi et al., 2011), wogonin (Takahashi et al., 2011), wogonoside (Wang et al., 2019), scutellarin (Ma et al., 2024; Zhou et al., 2022), chrysin (Wang et al., 2019), apigenin (Costine et al., 2022; Waheed et al., 2023), chlorogenic acid (Wan et al., 2021) and ferulic acid (Lu et al., 2011; El-Bassossy et al., 2016) (Figure 1). While metabolites like baicalein possess polyphenolic hydroxyl structures resembling pan-assay interference compounds (PAINS) and may cause false-positive signals *in vitro* (Bolz et al., 2021; Magalhães et al., 2021), substantial evidence supports the multi-mechanistic role of SB polyphenols in ischemic stroke, highlighting their therapeutic potential. To this end, this review aims to systematically elucidate the pharmacological mechanisms of SB polyphenols, providing a clear and reliable theoretical basis for future research.

## 3 Pharmacokinetics of polyphenolic metabolites from *Scutellaria baicalensis*

Studies using Sprague-Dawley (SD) rats as subjects revealed that the time to maximum plasma concentration (t_max_) of orally administered baicalein was 10.0 ± 0.0 min, whereas that of an equivalent dose of baicalin was significantly delayed to 395.6 ± 438.8 min (Lai et al., 2003). This discrepancy arises because baicalin requires hydrolysis by colonic microbiota before absorption, while baicalein is directly absorbed in the small intestine (Lai et al., 2003). Furthermore, in Wistar rats, the metabolic transformations of baicalin and baicalein exhibited marked differences. After oral administration of baicalin, baicalin itself was detectable in plasma, whereas free baicalein was nearly absent. Conversely, following oral administration of baicalein, plasma levels of baicalein were very low; however, baicalin appeared more rapidly than after direct baicalin administration (Akao et al., 2000). This may be attributed to the direct intestinal absorption of baicalein, followed by rapid metabolism via UDP-glucuronosyltransferase (UGT) into baicalin. In contrast, baicalin undergoes enterohepatic circulation: it is metabolized by gut microbiota to baicalein, which is reabsorbed and reconverted by UGT enzymes back into baicalin, re-entering systemic circulation (Tong et al., 2022). Additional studies reported a characteristic double-peak phenomenon in the plasma concentration-time curve of baicalin in SD rats due to enterohepatic recycling, with the time of the first maximum plasma drug concentration (t_max1_) at 0.20 ± 0.07 h and the second (t_max2_) at 5.60 ± 0.89 h (Zhu et al., 2010). The half-life of wogonin administered orally to SD rats was 27.97 ± 4.73 min, with a peak concentration of only 0.3 ± 0.08 mg/L and an oral bioavailability of 1.10% (Talbi et al., 2014). Another study indicated that the half-lives of wogonin in SD rats were 5.19 ± 0.51 h (oral) and 5.07 ± 0.64 h (intravenous), with an oral bioavailability of 1.37% ± 0.47% (Jeong et al., 2021). Although the reported half-lives differ significantly, the consistent low bioavailability underscores the challenge of oral absorption for wogonin. In comparison, wogonoside, the glucuronidated metabolite of wogonin, exhibited a half-life of 7.71 ± 1.55 h in SD rats and showed a double-peak phenomenon due to enterohepatic circulation, with t_max1_ at 0.17 ± 0.01 h and t_max2_ at 5.20 ± 1.80 h (Zhu et al., 2010; Sun et al., 2015). Pharmacokinetic studies of chrysin in SD rats reported an elimination half-life of 9.17 ± 3.16 h and a t_max_ of 5.20 ± 1.11 h (Dong et al., 2017). For scutellarin, the elimination half-life was 8.60 ± 0.90 h, with a t_max_ of 0.32 ± 0.02 h (Wang X. et al., 2021). In Wistar rats, apigenin had an elimination half-life of 7.87 ± 0.53 h and reached peak concentration at 3.60 ± 1.67 h (Kazi et al., 2020). SD rats administered chlorogenic acid exhibited an elimination half-life of 3.577 ± 0.474 h and a t_max_ of 0.250 ± 0.028 h (Yang et al., 2022a), while ferulic acid showed a half-life of 1.64 ± 0.66 h and a t_max_ of 0.097 ± 0.034 h (Zhu et al., 2020). Regarding tissue distribution, baicalein can cross the blood-brain barrier (BBB) and distribute uniformly across various brain regions (Tsai et al., 2002). Detectable levels of baicalin (Zhang et al., 2015), wogonin (Zhang et al., 2019), wogonoside (Zhang et al., 2019), apigenin (Zhang et al., 2019), and scutellarin (Liu X. et al., 2024) have been reported in the brain, suggesting their potential to penetrate the BBB and exert pharmacological effects. Ferulic acid also distributes into the brain, reaching a maximum concentration (180.354 ng/g) within 5 min, indicating that the brain may be a target organ for its action (An et al., 2021). In summary, the pharmacokinetic variability and tissue distribution profiles of polyphenolic metabolites from SB elucidate their molecular mechanisms of absorption, distribution, and metabolism. These findings provide critical insights into bioavailability limitations and inform targeted delivery strategies. Moreover, their ability to cross the BBB may underlie the therapeutic potential of SB polyphenols in the treatment of ischemic stroke.

## 4 Pharmacological effects of polyphenolic metabolites from *Scutellaria baicalensis*

**FIGURE 2:**
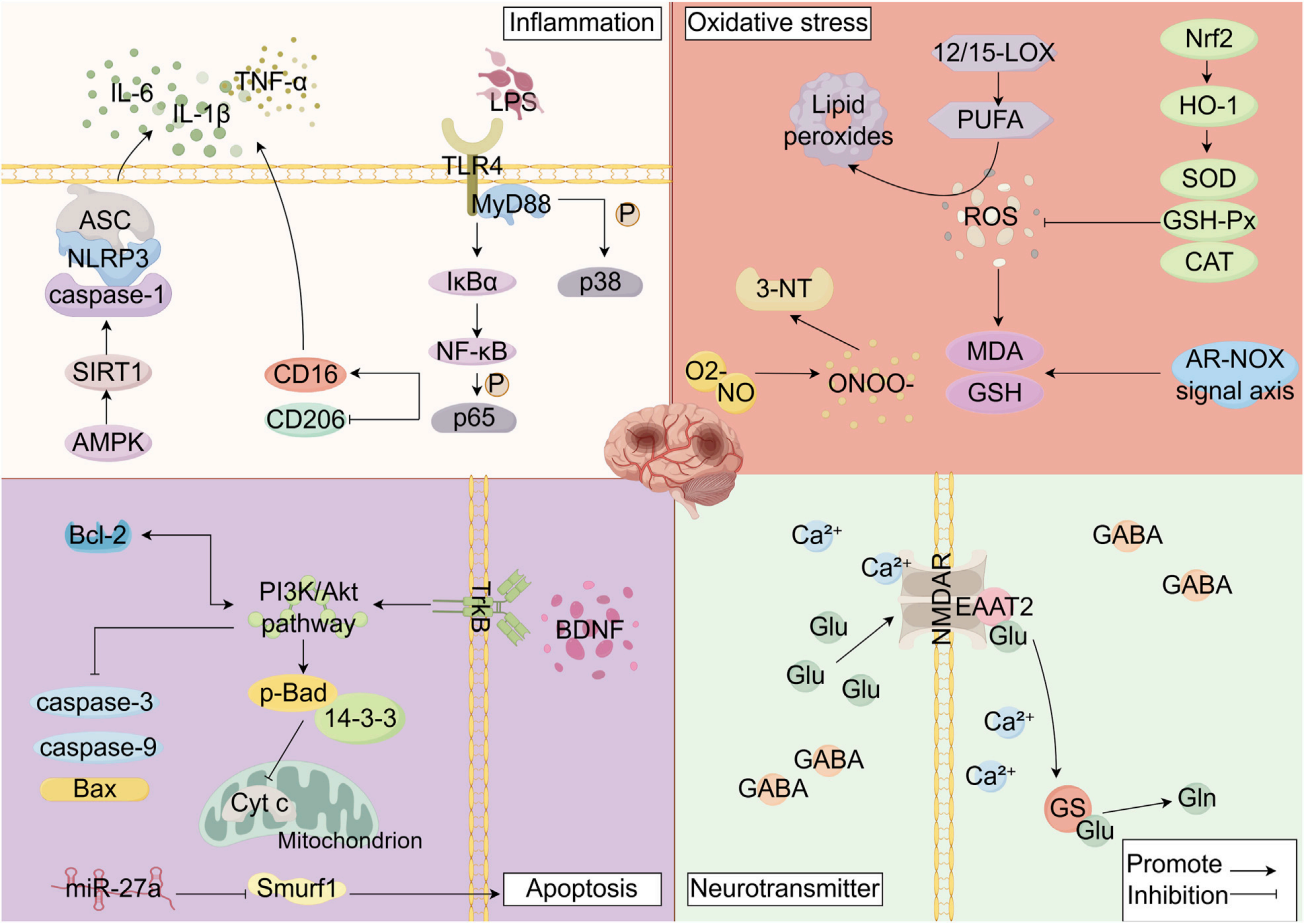
Mechanisms of Ischemic Stroke Injury (Interventional Mechanisms of *Scutellaria baicalensis* Polyphenolic Metabolites). By Figdraw. Note: LPS: Lipopolysaccharides, TLR4: Toll-like receptor 4, MyD88: myeloid differentiation primary response protein 88, IκBα: Inhibitor of nuclear factor kappa - B alpha, p38: p38 Mitogen-Activated Protein Kinase, NF-κB:Nuclear Factor kappa-B, P65: p65 Nuclear Factor Kappa B Subunit, CD16: Cluster of Differentiation 16, CD206: Cluster of Differentiation 206, AMPK: Adenosine 5′-monophosphate (AMP)-activated protein kinase, SIRT1: Sirtuin 1, caspase-1: Cysteine-dependent aspartate-directed protease-1, NLRP3: NOD-like receptor pyrin domain-containing protein 3, ASC: Apoptosis-associated speck-like protein containing a CARD, IL-6: Interleukin 6, IL-1β:Interleukin 1 beta, TNF-α: Tumor Necrosis Factor α, ROS: reactive oxygen species, 12/15-LOX:12/15-Lipoxygenase, PUFA: polyunsaturated fatty acids, MDA: malondialdehyde, GSH: glutathione, ONOO^−^: peroxynitrite, O_2-:_ Superoxide anion radical, NO: Nitric Oxide, 3-NT: 3-nitrotyrosine, Nrf2: Nuclear factor erythroid 2-related factor 2, HO-1: heme oxygenase-1, SOD: superoxide dismutase, GSH-Px: glutathione peroxidase, CAT: catalase, BDNF: Brain-derived neurotrophic factor, TrkB: tropomyosin receptor kinase B, p-BAD: Phosphorylated Bcl-2-associated death promoter, 14-3-3:14-3-3 proteins, Cyt **(C)** cytochrome c, Smurf1: Smad-specific E3 ubiquitin protein ligase 1, caspase-3: Cysteine-dependent aspartate-specific protease-3, caspase-9: Cysteine-dependent aspartate-specific protease-9, Bax: BCL-2-Associated X protein, Bcl-2: B-cell lymphoma 2 protein, miR-27a: microRNA-27a, NMDAR: N-methyl-D-aspartate receptor, Ca^2+^: Calcium ion, EAAT2: excitatory amino acid transporter 2, Glu: glutamate, Gln: glutamine, GS: glutamine synthetase, GABA: gamma-aminobutyric acid.

The polyphenolic metabolites of SB exert neuroprotective effects through multiple mechanisms. However, their structures suggest potential PAINS properties, which may lead to false-positive results in high-throughput screening assays via non-specific mechanisms. To circumvent such interference and ensure the accuracy of research conclusions, the studies cited in this review employ multi-tiered validation strategies to guarantee reliability. This is achieved by introducing targeted controls in in vitro models to exclude non-specific effects, using specific pathway inhibitors to confirm the core pathway-dependency of neuroprotection, and analyzing the activity of key metabolites in combination with *in vivo* stroke models. Ultimately, these approaches verify the authenticity of the pharmacological activity at a physiologically relevant level, ensuring the translatable potential of the experimental findings. This rigorous process not only secures the credibility of the mechanistic research on SB polyphenols but also lays an experimental foundation for their subsequent translation from basic research to clinical application. Extensive research has revealed that the research on polyphenolic metabolites derived from SB primarily focuses on baicalein, baicalin, wogonin, wogonoside, scutellarin, chrysin, apigenin, chlorogenic acid, and ferulic acid. These metabolites demonstrate neuroprotective effects against ischemic stroke through multi-target mechanisms, including anti-inflammatory, antioxidant, anti-apoptotic, regulation of neurotransmitter systems, maintenance of blood-brain barrier integrity, and inhibition of ferroptosis (Table 1; Figure 2).

**TABLE 1:** Polyphenolic metabolites from *Scutellaria baicalensis* in the treatment of ischemic stroke.

Active metabolite	Method	Dose	Model	Controls	Experiment duration	Targets	Actions	References
baicalein	*in vivo*	100 mg/kg	adult male C57BL/6J mice	1. Sham: sham operation + equal volume of CMC-Na 2. MCAO model: MCAO + equal volume of CMC-Na	72h	TLR4 p-IκBα p-p65 Iba-1 CD16 Arg-1 CD206 TNF-α IL-1β IL-6	anti-inflammatory	Ran et al. (2021)
	*in vivo*	30 mg/kg	male Sprague-Dawley rats	1. Sham: sham operation + equal volume of normal saline 2. Sham-vehicle: sham operation + equal volume of DMSO 3. MCAO model: MCAO + equal volume of normal saline 4. MCAO-vehicle: MCAO + equal volume of DMSO	24h	12/15-LOX p38 MAPK	Antioxidant	Cui et al. (2010)
	*in vivo*	300 mg/kg	male ALOX15 knockout mice wild-type C57BL/6J mice CD-1 mice	MCAO-vehicle: MCAO + equal volume of DMSO	24h	Claudin-5 IgG	maintenance of blood-brain barrier integrity	Jin et al. (2008)
	*in vivo*	100 mg/kg	male C57BL/6 mice	1. Sham: sham operation 2. MCAO/R model 3. MCAO/R + baicalein + sh-NC (5 × 10^8^ TU/ml, 2 μL) 4. MCAO/R + baicalein + sh-SIRT6 (5 × 10^8^ TU/ml, 2 μL) 5. MCAO/R + baicalein + sh-SIRT6+ ferrostatin-1 (2 mg/kg)	7d	SIRT6 SLC7A11 GPX4 GSH ACSL4 FOXA2	inhibition of ferroptosis	Fan et al. (2024)
baicalin	*in vivo*	10 mg/kg 50 mg/kg	ICR mice	1. Sham: sham operation 2. I/R + saline: I/R + normal saline	4h	TLR2/4 TNFα IL-1β NF-κB p65 MyD88	anti-inflammatory	Li et al. (2012)
	*in vitro*	5 μM 20 μM 50 μM	SH-SY5Y human neuroblastoma cells	1. SIN-1 group: SH-SY5Y + SIN-1 (1.5 mM) 2. ONOO^−^ group: SH-SY5Y + synthesized ONOO^−^ (50 μM)	24h	3-NT ONOO^−^	antioxidant	Xu et al. (2013)
	*in vitro*	34.38 μg/mL 8.59 μg/mL	co-culture of primary neurons and primary astrocytes isolated from Sprague-Dawley (SD) rats	OGD/R control: OGD/R (no extra intervention, standard medium)	221h	Bax caspase-3 caspase-9 Bcl-2	anti-apoptotic	Li et al. (2021a)
	*in vitro*	0.1 μmol/L 0.5 μmol/L 1 μmol/L 10 μmol/L 100 μmol/L	primary rat astrocytes isolated from the cerebral cortex of SD rats	1. OGD/R control: OGD/R 2. OGD/R + Malonate: OGD/R + Malonate (5 mmol/L)	24h	SDH ROS GS Glu Gln	regulation of neurotransmitter systems	Song et al. (2020)
wogonin	*in vivo*/*in vitro*	*in vivo*:20 mg/kg *in vitro*:0.1 μg/mL	*in vivo*:male SD rats *in vitro*:HT22 cells	*in vivo* 1. MCAO model: MCAO 2. Control: No MCAO (normal) *in vitro* 1. OGD/R control: OGD/R 2. OGD/R + Compound C: OGD/R + Compound C (10 μM)	*in vivo*: 24h *in vitro*:12h	TNF-α IL-1β IL-6 NLRP3 ASC cleaved caspase-1 IL-18	anti-inflammatory	Cheng et al. (2024)
wogonoside	*in vitro*	12.5 μM 25 μM 50 μM 100 μM 200 μM	PC12 cells	1. Control: Normal culture (no OGD/R) 2. OGD/R model: OGD/R	24h	HO-1 ROS SOD GSH MDA	antioxidant	Xu et al. (2024a)
	*in vitro*	12.5 μM 25 μM 50 μM 100 μM 200 μM	PC12 cells	1. Control: Normal culture (no OGD/R) 2. OGD/R model: OGD/R	24h	GABA	regulation of neurotransmitter systems	Xu et al. (2024a)
scutellarin	*in vivo*	40 mg/kg 80 mg/kg	male SD rats	1. Control: No ischemia (normal) 2. I/R model: I/R	24h	p-P38 p-P65	anti-inflammatory	Zhang et al. (2022)
	*in vivo*	50 mg/kg 100 mg/kg	male C57BL/6N mice	1. Sham: Sham operation 2. tMCAO + Vehicle: tMCAO +0.9% normal saline	72h	AR NOX1 NOX2 NOX4 ROS	antioxidant	Deng et al. (2022)
	*in vivo*	100 mg/kg	male SD rats	1. Sham: Sham operation 2. MCAO model: MCAO	72h	p-PI3K p-AKT Bcl-2 Bax activated caspase-3	anti-apoptotic	DUAN et al. (2025)
	*in vivo*	6 mg/kg 12 mg/kg	SD rats	1. Control: No MCAO (normal) 2. MCAO model: MCAO	12h	NMDA EAAT2 GABA Glu	regulation of neurotransmitter systems	Wang et al. (2023)
	*in vivo*	1.1505 mg/kg	male SD rats	1. Sham: Sham operation 2. Cerebral I/R model: Cerebral I/R injury 3. Positive (Nimodipine): Cerebral I/R + 0.5 mg/kg Nimodipine Injection 4. E. breviscapus injection: Cerebral I/R + 5 mL/kg E breviscapus injection 5. 3,5-dicaffeoylquinic acid: Cerebral I/R + 0.2335 mg/kg 3,5-dicaffeoylquinic acid	24h	MMP-9 claudin-5	maintenance of blood-brain barrier integrity	Liu et al. (2021a)
chrysin	*in vitro*	1 μM 5 μM 10 μM 20 μM 30 μM	PC12 cells	1. Control: Normal culture (no OGD/R) 2. OGD/R model: OGD/R	48h	PLAU p-NF-κB p-IκBα	anti-inflammatory	Li et al. (2022a)
apigenin	*in vivo*	60 mg/kg	male SD rats	1. Sham: Sham operation 2. MCAO model: MCAO 3. APG group: control +60 mg/kg Apigenin	14d	IL-1β IL-6	anti-inflammatory	Li et al. (2025b)
	*in vitro*	1 μM 10 μM 20 μM	PC12 cells	1. Control: Normal culture (no OGD/R) 2. OGD/R model: OGD/R	42h	Nrf2 HO-1 ROS SOD GSH-Px CAT	antioxidant	Guo et al. (2014)
chlorogenic acid	*in vivo*	5 mg/kg 10 mg/kg 15 mg/kg	Inbred male Charles foster albino rats	1. Sham: Sham operation 2. Ischemia model: Ischemia	8h	TNF-α iNOS	anti-inflammatory	Kumar et al. (2019)
	*in vivo*	30 mg/kg	male SD rats	1. PBS + Sham: Sham operation + PBS 2. CGA + Sham: Sham operation + Chlorogenic acid 3. PBS + MCAO: MCAO + PBS	24h	ROS MDA Trx	antioxidant	Kang et al. (2024a)
	*in vivo*	30 mg/kg	male Wistar rats	1. Sham: Sham operation 2. IR model: IR	24h	miR-27a Smurf1 TNF-α Bax Bcl-2	anti-apoptotic	Salimi et al. (2023)
	*in vitro*	10 μM 30 μM 50 μM	HT22 cells	1. Glu group: HT22 + 5 mM Glu 2. CGA group: HT22 + 10/30/50 μM CGA	24h	PP2A subunit B Glu	regulation of neurotransmitter systems	Kang et al. (2024b)
ferulic acid	*in vivo*	60 mg/kg 80 mg/kg 100 mg/kg	male SD rats	1. Sham: Sham operation 2. MCAO model: MCAO 3. DFA group: MCAO +100 mg/kg Ferulic acid (i.v., 30 min post-MCAO)	24h	ICAM-1 MPO NF-κb p50	anti-inflammatory	Cheng et al. (2008)

TLR4: Toll - like receptor 4, p-IκBα: phosphorylated Inhibitor of nuclear factor kappa - B, alpha, p-p65: Phosphorylated nuclear factor kappa-B p65 subunit, Iba-1: Ionized calcium - binding adapter molecule 1, CD16: Cluster of Differentiation 16, Arg-1: Arginase 1, CD206: Cluster of Differentiation 206, TNF-α: Tumor Necrosis Factor–alpha, IL-1β: Interleukin - 1 beta, IL-6: Interleukin – 6, 12/15-LOX: 12/15-Lipoxygenase, p38 MAPK: p38 mitogen - activated protein kinase, Claudin-5: Claudin-5, IgG: Immunoglobulin G, SIRT6: Silent Information Regulator 6, SLC7A11: Solute Carrier Family 7 Member 11, GPX4: Glutathione Peroxidase 4, GSH: glutathione, ACSL4: Long-chain acyl-coenzyme A synthetase family Member 4, FOXA2: Forkhead Box Protein A2, TLR2: Toll - like receptor 2, NF-κB p65: Nuclear Factor-Kappa B p65 subunit, MyD88: Myeloid differentiation primary response protein 88, 3-NT: 3-Nitrotyrosine, ONOO^−^: peroxynitrite, Bax: BCL-2-Associated X protein, caspase-3: Cysteine-dependent aspartate-specific protease-3, caspase-9: Cysteine-dependent aspartate-specific protease-9, Bcl-2: B-cell lymphoma 2 protein, SDH: succinate dehydrogenase, ROS: reactive oxygen species, GS: glutamine synthetase, Glu: glutamate, Gln: glutamine, NLRP3: Nucleotide-binding oligomerization domain-like receptor family pyrin domain containing 3, ASC: Apoptosis-associated speck-like protein containing a CARD, cleaved caspase-1: Cleaved Cysteine-dependent aspartate-directed protease-1, IL-18: Interleukin – 18, HO-1: Heme Oxygenase-1, SOD: superoxide dismutase, MDA: malondialdehyde, GABA: gamma-aminobutyric acid, p-P38: Phosphorylated p38 Mitogen-Activated Protein Kinase, AR: aldose reductase, NOX1: Nicotinamide Adenine Dinucleotide Phosphate Oxidase 1, NOX2: Nicotinamide Adenine Dinucleotide Phosphate Oxidase 2, NOX4: Nicotinamide Adenine Dinucleotide Phosphate Oxidase 4, p-PI3K: Phosphorylated Phosphoinositide 3-Kinase, p-AKT: Phosphorylated Protein Kinase B, activated caspase-3: Activated Cysteine-dependent aspartate-specific protease-3, NMDA: N-methyl-D-aspartate receptor, EAAT2: Excitatory Amino Acid Transporter 2, MMP-9: Matrix Metalloproteinase-9, PLAU: plasminogen activator, Urokinase, p-NF-κB: Phosphorylated Nuclear Factor-kappa B, Nrf2: Nuclear factor erythroid 2-related factor 2, GSH-Px: Glutathione Peroxidase, CAT: catalase, iNOS: inducible nitric oxide synthase, Trx: thioredoxin, miR-27a: microRNA-27a, Smurf1: Smad-specific E3 ubiquitin protein ligase 1, PP2A subunit B: Protein Phosphatase 2A Regulatory Subunit B, ICAM-1: Intercellular Cell Adhesion Molecule-1, MPO: myeloperoxidase, NF-κb p50: Nuclear Factor kappa-light-chain-enhancer of activated B cells 1 p50, CMC-Na: carboxymethylcellulose sodium solution, DMSO: dimethyl sulfoxide, PBS: phosphate buffered saline, APG: apigenin, CGA: chlorogenic acid, MCAO: middle cerebral artery occlusion, tMCAO: transient Middle Cerebral Artery Occlusion, OGD/R: oxygen-glucose deprivation/reperfusion.

### 4.1 The anti-inflammatory effects

Inflammation serves as a fundamental pathological process underlying the damage in ischemic stroke. Pattern recognition receptors, such as Toll-like receptors (TLRs), are crucial regulators of the inflammatory response in ischemia/reperfusion injury (Yang et al., 2021). Under conditions of ischemic/reperfusion injury, toll-like receptor 4 (TLR4) is activated and facilitates the recruitment of myeloid differentiation primary response protein 88 (MyD88) (Zhang X. et al., 2017). Interleukin-1 Receptor-Associated Kinase 4 (IRAK4) interacts directly with MyD88, and activated IRAK4 is recruited to TNF Receptor Associated Factor 6 (TRAF6). This subsequently leads to the activation of the Inhibitor of kappaB Kinase (IKK) signaling cascade via Transforming Growth Factor-β-Activated Kinase 1 (TAK1), ultimately resulting in the activation of the Nuclear Factor kappa-B (NF-κB) pathway (Kang et al., 2023), the activation of NF-κB directly induces the production of pro-inflammatory cytokines, including Tumor Necrosis Factor (TNF)-α and Interleukin (IL)-6 (Ciesielska et al., 2021). Furthermore, TLR4 signaling contributes to the activation of the NLRP3 inflammasome, promoting the cleavage of pro-IL-1β and pro-IL-18 by Cysteine-dependent aspartate-directed protease-1 (caspase-1) into their active forms, IL-1β and IL-18 (Ciesielska et al., 2021; Xu S. et al., 2024). Additionally, the Mitogen-Activated Protein Kinase (MAPK) pathway, which can be activated by TAK1, stimulates p38, ERK, and JNK, thereby fostering the production of pro-inflammatory cytokines such as TNF-α and IL-6 and mediating the inflammatory response (Zhang P. et al., 2020). Numerous studies have demonstrated that the activation of microglia and astrocytes plays a central role in mediating neuroinflammation (Liu M. et al., 2020). Microglia can polarize into the pro-inflammatory M1 phenotype, often associated with increased expression of ionized calcium-binding adapter molecule 1 (Iba-1), or the anti-inflammatory M2 phenotype (Jiang et al., 2020; Yu et al., 2022). Similarly, astrocytes can adopt a neurotoxic A1 phenotype, characterized by elevated expression of Glial Fibrillary Acidic Protein (GFAP), or a neuroprotective A2 phenotype (Xu D. et al., 2021; Liang et al., 2023). During the early stages of ischemic stroke, activated pro-inflammatory microglia can disrupt gap junctions and enhance the permeability of connexin 43 (Cx43) hemichannels on astrocytes through the release of pro-inflammatory factors, conversely, astrocytes can promote the polarization of microglia towards a pro-inflammatory phenotype via their Cx43 hemichannels, creating a vicious cycle that exacerbates the neuroinflammatory cascade and amplifies damage following ischemic stroke (Liang et al., 2023). It is noteworthy that pericytes surrounding cerebral microvessels contribute to the pro-inflammatory response by generating TLR4 after ischemic stroke (Alsbrook et al., 2023). A1 astrocytes participate in cerebral ischemia-induced neuroinflammation through the TLR4/NF-κB signaling pathway (Liu R. et al., 2024). As primary cellular expressers of TLR4, activated microglia further induce the infiltration of inflammatory cells and the production of cytokines, adhesion molecules, chemokines, and other inflammatory mediators. This process promotes the accumulation and infiltration of neutrophils into the ischemic area, ultimately establishing a persistent vicious cycle of inflammation. Collectively, these pathological processes lead to the disruption of the BBB and exacerbate secondary neuronal apoptosis (Liu M. et al., 2020; Alsbrook et al., 2023).

Anti-inflammatory effects of baicalein in ischemic stroke models via multiple pathways. Studies have demonstrated that baicalein exerts anti-inflammatory effects in models of ischemic stroke through multiple mechanisms. It significantly reduces serum levels of IL-6, IL-1β, and TNF-α in mouse models of ischemic stroke, thereby attenuating systemic inflammation post-stroke (Zhang LK. et al., 2025). Mechanistically, in Middle Cerebral Artery Occlusion (MCAO) models, baicalein modulates the TLR4/NF-κB pathway by reducing microglial TLR4 expression, inhibiting IKBα and p65 phosphorylation, and decreasing p65 nuclear translocation. Consequently, it downregulates mRNA expression of the pro-inflammatory marker CD16 while upregulating the anti-inflammatory marker CD206 (Ran et al., 2021). Additionally, baicalein significantly suppresses phosphorylation of JNK, ERK, and P38 proteins via the MAPK signaling pathway in MCAO rat models, thereby reducing neuroinflammatory signaling and brain injury induced by ischemic stroke (Yang et al., 2019). Baicalin also targets the TLR4/NF-κB signaling pathway to exert anti-inflammatory effects. In oxygen-glucose deprivation (OGD)-induced PC12 cells, baicalin specifically targets TLR4, downregulating TLR4 and MyD88 expression, blocking p65 nuclear translocation, and thereby inhibiting downstream NF-κB pathway activation. This leads to reduced release of TNF-α and IL-1β (Li et al., 2012). *In vivo* studies further confirmed that in a mouse model of ischemia/reperfusion (I/R) injury, baicalin significantly decreases TLR4 expression in the hippocampus and inhibits production of inflammatory mediators such as TNF-α and IL-1β, supporting its neuroprotective role via the TLR4/NF-κB pathway (Li et al., 2012). In Middle Cerebral Artery Occlusion/Reperfusion (MCAO/R) models, baicalin markedly reduces expression of the pro-inflammatory microglial marker CD16 and enhances expression of the anti-inflammatory microglial marker CD206 (Wang et al., 2024; Zhang S. et al., 2025). Moreover, baicalin inhibits astrocyte activation, as evidenced by significantly reduced GFAP expression in astrocytes and decreased release of IL-6, IL-1β, and TNF-α in the brain tissue of transient Middle Cerebral Artery Occlusion (tMCAO) mice (Li YF. et al., 2025). Baicalin also suppresses pro-inflammatory enzymes, including inducible nitric oxide synthase (iNOS) and cyclooxygenase-2 (COX-2), in MCAO rat models (Zhang Q. et al., 2017), both of which are strongly associated with inflammatory responses in ischemic stroke (Li T. et al., 2022; Li L. et al., 2021). Scutellarin exerts anti-inflammatory effects by modulating the polarization of microglia and astrocytes. *In vitro*, scutellarin significantly reduces protein expression levels of TNF-α and IL-6 released by LPS (Lipopolysaccharides)-activated microglia (Ye et al., 2023; Yuan et al., 2015; Duan et al., 2024). *In vivo*, scutellarin lowers protein expression of P-IκBα and P-P65 in astrocytes of the cortex in MCAO mouse models, inhibiting NF-κB pathway activation and preventing polarization of astrocytes toward the neurotoxic A1 phenotype (Zou et al., 2024). Additionally, scutellarin regulates the MAPK/NF-κB in MCAO rat models, reducing levels of p-P38 and p-P65 in a dose-dependent manner to protect the brain from ischemic injury (Zhang et al., 2022). In MCAO rat models, chlorogenic acid exhibits neuroprotective and anti-inflammatory effects by modulating glial cell polarization. It downregulates Iba-1 protein expression in the ischemic cortex, inhibiting microglial activation (Shah et al., 2022a), and reduces GFAP levels in astrocytes, thereby attenuating astrocyte activation induced by ischemic injury (Shah et al., 2022a). Furthermore, in BCCAO (Bilateral Common Carotid Artery Occlusion) rat models, chlorogenic acid significantly decreases TNF-α expression in the ischemic cortex, demonstrating anti-inflammatory efficacy (Kumar et al., 2019). The anti-inflammatory effects of ferulic acid are time-dependent. In the early hours of cerebral ischemic injury, infiltrating leukocytes release pro-inflammatory mediators (Xu Y. et al., 2021), a process dependent on Intercellular Cell Adhesion Molecule-1 (ICAM-1) for leukocyte recruitment to inflammatory sites (Wang L. et al., 2021). In MCAO rat models, ferulic acid reduces ICAM-1 expression in the striatum after 2 h of reperfusion, decreasing leukocyte adhesion. After 24 h of reperfusion, it reduces myeloperoxidase (MPO)-positive cells and NF-κB activation in the cortex, thereby interrupting the inflammatory cascade and mitigating damage (Cheng et al., 2008). Wogonin significantly inhibits expression of pro-inflammatory cytokines TNF-α, IL-1β, and IL-6 in MCAO rat models, alleviating neuroinflammation after ischemia-reperfusion injury (Cheng et al., 2024), *in vitro* studies using oxygen-glucose deprivation/reperfusion (OGD/R)-induced HT22 cells show that wogonin activates the AMPK/SIRT1 signaling axis, downregulating protein levels of NLRP3, ASC (Apoptosis-associated speck-like protein containing a CARD), cleaved caspase-1, and IL-18, thereby inhibiting inflammasome assembly and activation (Cheng et al., 2024). Chrysin exerts anti-inflammatory effects in ischemic stroke by targeting plasminogen activator urokinase (PLAU) and inactivating the NF-κB pathway. In OGD/R-induced PC12 cells, chrysin downregulates PLAU expression and suppresses phosphorylation of NF-κB and IκBα, blocking inflammatory signaling and attenuating the inflammatory cascade (Li N. et al., 2022). Additionally, chrysin reduces IL-1β and TNF-α protein levels in the hippocampus of I/R rat models, mitigating neuroinflammation and protecting against brain injury (Sarkaki et al., 2019; Khombi Shooshtari et al., 2021). Apigenin not only reduces protein levels of IL-1β and IL-6 in the ischemic penumbra of MCAO rat models but also modulates gut microbiota, contributing to comprehensive anti-inflammatory effects and maintenance of intestinal homeostasis, thereby ameliorating cerebral ischemic injury (Li J. et al., 2025).

### 4.2 The antioxidant effects

Oxidative stress results from an imbalance between the sustained generation of reactive oxygen species (ROS) or free radicals and their clearance by antioxidant mechanisms (Pawluk et al., 2024; Jelinek et al., 2021). ROS are considered byproducts of energy metabolism during cellular activities (Yang and Lian, 2020). Under physiological conditions, redox homeostasis is maintained as ATP synthesis produces ROS and redox enzymes eliminate the excess (Yang and Lian, 2020; Herb and Schramm, 2021). However, under pathological conditions, the brain is particularly vulnerable to oxidative stress owing to its high oxygen consumption, abundance of polyunsaturated fatty acids (PUFAs) in membrane lipids, and relatively limited antioxidant defense capacity, neuronal redox signaling acts as an intrinsic sensor of oxidative stress (Trofin et al., 2025; Lee et al., 2020). 12/15-Lipoxygenase (12/15-LOX) plays a critical role in catalyzing the oxidation of PUFAs, promoting the formation of lipid peroxides (Jiang et al., 2013; Wang et al., 2025), thereby exacerbating oxidative brain damage. In ischemic stroke, oxidative stress is closely associated with cerebral pathological changes. The ischemic brain, characterized by high aerobic metabolism, perfusion demands, and relatively weak antioxidant defenses, is highly susceptible to oxidative damage induced by excessive ROS levels. This is accompanied by the activation of pro-oxidant enzyme systems such as NADPH oxidase (NOX), which catalyzes the overproduction of superoxide anion (O_2_^−^) (Liu M. et al., 2021; Chen et al., 2011). Concurrently, ischemic stroke leads to elevated levels of lipid peroxidation markers such as malondialdehyde (MDA), while the activities of antioxidant enzymes responsible for ROS degradation, including superoxide dismutase (SOD), glutathione peroxidase (GSH-Px), and catalase (CAT), are significantly reduced, indicating intensified oxidative stress (Kamal et al., 2023). Moreover, in ischemic brain tissue, O_2_^−^ reacts with nitric oxide (NO) to form peroxynitrite (ONOO^−^), a well-defined cytotoxic agent in ischemic brain injury (Gong et al., 2015). In summary, oxidative stress contributes to cerebral pathologies in ischemic stroke, including disruption of cellular homeostasis, neuronal death, and structural damage to ischemic brain tissue (Godínez-Rubí et al., 2013).

Numerous studies have demonstrated that baicalein exhibits significant antioxidant and neuroprotective effects across various experimental models. Iodoacetic acid (IAA) can mimic hypoxic/ischemic conditions in neural cells *in vitro*, leading to increased ROS levels and subsequent cell death (Zhou et al., 2015). Baicalein, at concentrations ranging from 2.5 to 10 μM, significantly counteracted the oxidative stress cascade induced by IAA in cultured HT22 mouse hippocampal cells, increasing cell viability by approximately 80% (Lapchak et al., 2007). This antioxidant property was further validated in an OGD/R-induced HT22 cell models, where baicalein effectively reduced intracellular ROS and O_2_^−^ levels, mitochondrial superoxide, and malondialdehyde (MDA) content, while increasing glutathione (GSH) levels (Fan et al., 2024; Li M. et al., 2022). In MCAO models, baicalein not only significantly reduced the mRNA and protein expression levels of 12/15-lipoxygenase (12/15-LOX) in the ischemic cortex but also decreased cell death in glutamate-treated oxidative-stressed primary cortical neurons (Cui et al., 2010; Van Leyen et al., 2006). Notably, the inhibitory effect of baicalein on 12/15-LOX exerted multiple protective benefits, including reduced lactate dehydrogenase release and protection of human brain endothelial cells against oxidative damage (Jin et al., 2008). These findings collectively indicate that baicalein exerts neuroprotective effects by modulating oxidative stress-related pathways. NADPH oxidase (NOX), which produces superoxide, plays a critical role in the pathophysiology of ischemic stroke (Kim et al., 2017). Aldose reductase (AR), a key enzyme involved in oxidative stress, regulates NOX isoforms such as NOX2, NOX1, and NOX4 following ischemic stroke (Deng et al., 2022; Li et al., 2024). In tMCAO mouse models, scutellarin was shown to modulate the AR–NOX signaling axis, downregulating both mRNA and protein expression levels of AR and NOX isoforms (NOX2, NOX1, and NOX4), thereby reducing the accumulation of oxidative damage markers (Deng et al., 2022). These results suggest that scutellarin alleviates ischemic stroke injury by targeting the AR–NOX signaling axis to regulate oxidative stress. In the SH-SY5Y cell models, 3-nitrotyrosine (3-NT) serves as a biomarker for ONOO^−^ formation. Baicalin dose-dependently inhibited 3-NT generation induced by the ONOO^−^ donor SIN-1, effectively attenuating both ONOO^−^ mediated cytotoxicity and cell death caused by OGD/R (Xu et al., 2013). This protective effect was further confirmed in MCAO/R rat models, where baicalin reduced ONOO^−^ levels and ameliorated ischemic stroke injury (Chen et al., 2018). Nuclear factor erythroid 2-related factor 2 (Nrf2) is a key regulator of the endogenous antioxidant response. Activation of Nrf2 upregulates the expression of various antioxidant enzymes, including NAD(P)H:quinone oxidoreductase 1 (NQO-1), heme oxygenase-1 (HO-1), superoxide dismutase (SOD), and glutathione peroxidase (GSH-Px), thereby mitigating cerebral oxidative stress (Huang et al., 2021). In tMCAO rat models, baicalin modulated the Nrf2/HO-1 pathway, significantly upregulating the expression of Nrf2, SOD, GSH-Px, HO-1, and NQO-1 in the ischemic brain region, this led to suppressed ROS accumulation and protection against neuronal ischemic injury (Huang et al., 2021). In OGD/R-induced PC12 cell models, apigenin significantly reduced ROS levels, upregulated the expression of SOD, GSH-Px, and catalase (CAT), and markedly increased both Nrf2 expression and HO-1 mRNA levels, effectively alleviating oxidative stress injury (Guo et al., 2014). Similarly, wogonoside enhanced SOD and GSH activity, decreased MDA levels, and activated the Nrf2/Sirt3 pathway in OGD/R-induced PC12 cell models. It upregulated the antioxidant enzyme HO-1, thereby promoting ROS clearance and reducing oxidative damage (Xu D. et al., 2024). Sirt3, a member of the sirtuin family, contributes to antioxidant defense. NRF2 acts as a novel regulator of SIRT3 by directly binding to its promoter and increasing its expression, ultimately attenuating oxidative stress (Hu et al., 2024; Ge et al., 2024). Thioredoxin (Trx) and ubiquitin C-terminal hydrolase L1 (UCH-L1) are key antioxidant proteins that play crucial roles in counteracting oxidative stress and conferring neuroprotection (Ohmori et al., 2022; Liu et al., 2011). Chlorogenic acid was found to inhibit the decrease in Trx and UCH-L1 expression in the ischemic cortex of MCAO rat models, thereby alleviating oxidative stress damage following ischemic stroke (Kang et al., 2024a; Shah et al., 2022b). Additionally, chlorogenic acid significantly reduced ROS and lipid peroxide (LPO) levels in the MCAO rat models, demonstrating antioxidant efficacy in mitigating ischemic stroke injury (Shah et al., 2023).

### 4.3 The anti-apoptotic effects

Apoptosis, a form of programmed cell death, maintains dynamic equilibrium in brain tissue under physiological conditions by balancing cell death and proliferation. However, following ischemic stroke, apoptosis markedly increases and becomes a major cause of neuronal loss (Xu Z. et al., 2024; Li L. et al., 2023). Apoptosis is regulated through multiple pathways, among which the polyphenolic metabolites of SB primarily inhibit neuronal apoptosis via the Phosphoinositide 3-kinase (PI3K)/AKT signaling pathway, thereby exerting protective effects against neuronal damage in ischemic stroke.Under cerebral ischemic stress, the expression of the anti-apoptotic protein Bcl-2 decreases, while that of the pro-apoptotic protein Bax increases. This imbalance leads to activation of the mitochondrial apoptotic cascade, with the cleavage of Caspase-1 and Caspase-3 playing pivotal roles in the early phases of ischemia-mediated apoptosis (Liu X. et al., 2022). Extracellular signals such as ischemia can activate the PI3K/AKT pathway. Once activated, AKT phosphorylates the pro-apoptotic protein Bad, facilitating its binding to 14-3-3 proteins. This interaction increases the availability of free Bcl-2 or Bcl-xL, promoting cell survival. Additionally, activated AKT enhances Bcl-2 levels through multiple indirect mechanisms, contributing directly to the suppression of apoptosis (Liu T. et al., 2025).

Brain-derived neurotrophic factor (BDNF) is not only produced in neurons but is also significantly secreted by astrocytes (Hong et al., 2016). Its pro-survival and neuroprotective functions are primarily mediated through the PI3K/Akt signaling pathway, which is activated upon binding to the tropomyosin receptor kinase B (TrkB) (Liu W. et al., 2020). Studies have shown that baicalin promotes BDNF secretion in OGD/R neuron-astrocyte co-culture models. This leads to activation of the TrkB receptor and its downstream PI3K/Akt pathway, triggering a signaling cascade that upregulates Bcl-2 protein expression while suppressing Bax, caspase-3, and caspase-9 levels, thereby attenuating neuronal apoptosis (Li C. et al., 2021; Liu W. et al., 2020). In rat models of I/R injury, apoptotic cells are widely observed in brain tissue. Scutellarin significantly reduced the number of TUNEL-positive cells and the percentage of apoptotic cells in the ischemic cortex of MCAO rat models (Yang C. et al., 2022). This anti-apoptotic effect is associated with enhanced phosphorylation of PI3K and AKT, upregulation of Bcl-2, facilitation of PI3K/AKT signaling transduction, and downregulation of Bax and activated caspase-3 (Duan et al., 2025). Chlorogenic acid exerts anti-apoptotic effects through multiple mechanisms. First, it activates the PI3K/Akt pathway, reversing the decreased expression of p-PDK1, p-Akt, and p-Bad in the cerebral cortex of MCAO rat models. It promotes the binding of phosphorylated Bad to 14-3-3 protein, thereby inhibiting Bad’s pro-apoptotic function and preventing cytochrome c (Cyt c) release from mitochondria, which blocks the apoptotic cascade (Shah et al., 2022c). Second, chlorogenic acid modulates apoptosis via the microRNA (miR)-27a/Smurf1 pathway. miR-27a promotes apoptosis, while Smad-specific E3 ubiquitin protein ligase 1 (Smurf1) suppresses it (Li et al., 2019; Fu et al., 2020). As a downstream target of miR-27a, Smurf1 influences apoptotic activity (Zhao et al., 2020). Chlorogenic acid significantly downregulates miR-27a expression in the cortex of common carotid artery occlusion (CCAO) rat models, alleviating its repression of Smurf1 and restoring Smurf1’s anti-apoptotic function. Additionally, it reduces the release of the inflammatory factor TNF-α, thereby inhibiting neuroinflammation-mediated apoptosis (Salimi et al., 2023). Finally, chlorogenic acid directly decreases Bax expression, upregulates Bcl-2, lowers the Bax/Bcl-2 ratio, and directly exerts anti-apoptotic effects (Salimi et al., 2023).

### 4.4 The regulation of neurotransmitter action

Neurotransmitters are chemical substances that transmit signals between neurons or from neurons to effector cells. Over 200 distinct neurotransmitters have been identified to date (Yang Y. et al., 2024; Teleanu et al., 2022). Current researches on the neuroprotective effects of SB polyphenols against ischemic stroke-induced neuronal injury has primarily focused on their modulation of glutamate and gamma-aminobutyric acid (GABA). Glutamate is an essential excitatory neurotransmitter in the nervous system, playing a critical role in maintaining basic brain functions and contributing significantly to the development of the central nervous system (CNS) (Nimgampalle et al., 2023). However, excessive release of glutamate can lead to excitotoxicity (Nimgampalle et al., 2023). This excitotoxic effect is largely mediated through the N-methyl-D-aspartate receptor (NMDAR). Cerebral ischemia triggers a massive release of glutamate. The excessive activation of NMDAR, the most calcium-permeable ionotropic glutamate receptor, induces calcium influx, thereby exacerbating excitotoxicity and serving as a primary cause of neuronal death in ischemic stroke (Lai et al., 2014; Zong et al., 2022; Kang et al., 2022). It is noteworthy that excitatory amino acid transporter 2 (EAAT2), a major glutamate transporter, facilitates the uptake of glutamate from the synaptic cleft, thereby preventing its abnormal accumulation and mitigating excitotoxic damage (Das et al., 2025). GABA serves as the principal inhibitory neurotransmitter in the CNS, functioning to prevent neuronal overexcitation and coordinate neuronal activity (Rodrigues et al., 2024; Stragie et al., 2017). Importantly, GABA counteracts the excitotoxic effects of glutamate and enhances neuronal tolerance to ischemic conditions (Liu BH. et al., 2022).

In the OGD/R-induced primary rat astrocyte models, baicalin suppresses mitochondrial ROS overproduction by inhibiting succinate dehydrogenase (SDH) activity. Concurrently, it protects glutamine synthetase (GS) from 20S proteasomal degradation, thereby preserving GS protein stability and catalytic function, activated GS utilizes glutamate as a substrate to synthesize glutamine, which in turn enhances astrocytic uptake of synaptic glutamate, effectively preventing extracellular glutamate accumulation and mitigating excitotoxic cascades (Song et al., 2020). Scutellarin regulates glutamatergic signaling through dual mechanisms. In the MCAO rat models, it suppresses excessive activation of NMDARs and upregulates EAAT2 expression in the ischemic cortex and hippocampus, thereby rebalancing the concentrations of glutamate, GABA, and phenylalanine and reducing excitotoxic damage (Wang et al., 2023). Additionally, in OGD-induced hippocampal neuronal models, scutellarin reduces intracellular calcium concentrations, attenuating calcium overload and further protecting neurons from injury (Dang et al., 2019). Chlorogenic acid exerts neuroprotective effects by targeting protein phosphatase 2A (PP2A) and calcium homeostasis. In glutamate-treated HT22 cell models, it reverses the downregulation of the PP2A subunit B, thereby preventing enhanced excitotoxicity due to reduced PP2A activity (Kang et al., 2024b). In BCCAO rat models, chlorogenic acid significantly reduces calcium and glutamate concentrations in the cerebral cortex, cerebellum, hippocampus, and cerebrospinal fluid, alleviating excitotoxicity by inhibiting calcium overload and abnormal glutamate accumulation (Kumar et al., 2019). Wogonoside significantly restores decreased GABA levels in OGD/R-induced PC12 cell models to near-normal physiological conditions, thereby inhibiting neuronal hyperexcitability and reducing excitotoxicity-related damage (Xu D. et al., 2024).

### 4.5 Other effects

BBB disruption is a critical adverse event following ischemic stroke, often leading to severe malignant cerebral edema (Li Y. et al., 2023; Qiu et al., 2021). The expression and activity of matrix metalloproteinase-9 (MMP-9) are markedly upregulated within hours after cerebral ischemia, resulting in reduced endothelial tight junctions and ultimately BBB breakdown (Ji et al., 2023). Claudin proteins are key structural metabolites of tight junctions, with claudin-5 being particularly crucial for maintaining BBB integrity (Greene et al., 2022). In rat models of I/R injury, Scutellarin was shown to protect against BBB damage by inhibiting MMP-9 transcription and synthesis, while upregulating claudin-5 protein expression (Liu G. et al., 2021). Similarly, baicalein attenuated Claudin-5 degradation in the brains of MCAO mouse models, thereby preserving BBB integrity and reducing leakage and ischemic edema (Jin et al., 2008). Notably, post-stroke BBB disruption can further perturb iron metabolism and compromise the antioxidant system (Tian et al., 2024). Ferroptosis, a key mechanism in ischemic stroke, is primarily driven by iron-dependent lipid peroxidation. This process involves Fe^2+^ or lipoxygenase (LOX)-catalyzed peroxidation of abundantly expressed PUFAs in cell membranes, ultimately leading to cell death (Deng et al., 2023). Glutathione peroxidase 4 (GPX4) serves as a key inhibitor of ferroptosis by utilizing GSH to eliminate lipid peroxides, thus blocking the execution of ferroptosis (Liu J. et al., 2024; Zhang et al., 2021). Among the long-chain acyl-coenzyme A synthetase (ACSL) family, ACSL3 enhances resistance to ferroptosis, whereas ACSL4 acts as a specific biomarker and driver of this process (Yang et al., 2022c). Baicalein effectively counteracts the inhibitory effect of RAS-selective lethal small molecule 3 (RSL3), a known GPX4 inhibitor (Li S. et al., 2021). In RSL3-stimulated HT22 cell models, baicalein modulated the GPX4/ACSL4/ACSL3 axis by upregulating GPX4 and ACSL3 expression while suppressing ACSL4, thereby inhibiting ferroptosis (Li M. et al., 2022). Additionally, silent information regulator 6 (SIRT6), an NAD^+^-dependent deacetylase, downregulates forkhead box protein A2 (FOXA2) at both expression and acetylation levels, reducing its transcriptional activity. This indirectly upregulates solute carrier family 7 member 11 (SLC7A11), effectively suppressing ferroptosis (Fan et al., 2024; Zhang W. et al., 2017). In MCAO/R mouse models, baicalein upregulated SIRT6 expression, inhibited FOXA2-mediated transcriptional repression, and cooperatively enhanced SLC7A11 and GPX4 levels. This led to increased GSH biosynthesis and reduced ACSL4 expression, multi-targetly inhibiting ferroptosis (Fan et al., 2024). Furthermore, ischemic stroke is often accompanied by a hypercoagulable state that promotes thrombosis. Baicalin demonstrated antiplatelet aggregation and pro-circulatory activities in MCAO rat models, thereby attenuating secondary damage through its antithrombotic effects (Liu H. et al., 2021).

## 5 Strategies to enhance the bioavailability of *Scutellaria baicalensis* polyphenols

Polyphenols, as neuroprotective agents, can directly act on CNS cells and processes to improve brain function. This requires that sufficient quantities of polyphenols must cross the BBB and reach brain tissue in their active form (Grabska-Kobyłecka et al., 2023). However, their therapeutic efficacy is often limited by low selective permeability across the BBB, poor absorption, rapid metabolism, and high systemic clearance, all of which reduce their bioavailability (Pandareesh et al., 2015). To overcome these limitations, nano-encapsulation technologies have been developed to enhance the bioavailability of polyphenolic metabolites (Yang et al., 2020). Various nano-delivery systems, such as polymeric micelles, liposomes, and polymeric nanoparticles, can effectively encapsulate polyphenols, improving their stability, absorption, and targeted delivery to the brain.

Poly (ethylene glycol)-block-poly (D,L-lactide) (PEG-PLA), an amphiphilic diblock copolymer, can be used to encapsulate baicalein into micelles. Following intranasal administration in mice, these micelles significantly enhanced both the bioavailability of baicalein in plasma and its distribution to the brain (Zhang L. et al., 2020). Liposomes loaded with baicalin provide sustained and controlled release *in vitro*, prolonging its duration of action. Intranasal delivery bypasses the BBB, allowing direct brain entry and avoidance of first-pass metabolism. This strategy markedly improved neurological function, reduced cerebral infarct volume, and alleviated pathological damage in the CA1 region of the hippocampus in MCAO rat models (Yu et al., 2023). Additionally, D-α-tocopheryl polyethylene glycol succinate (TPGS), a water-soluble derivative of vitamin E and PEG 1000, was used to formulate chlorogenic acid-loaded liposomes. Oral administration in SD rats resulted in a 1.52-fold increase in the bioavailability of chlorogenic acid compared to the unformulated drug (Zhang et al., 2024). Poly (lactic-co-glycolic acid) (PLGA), a copolymer of lactic acid and glycolic acid, was employed to prepare scutellarin-loaded PLGA nanoparticles (SCU-PLGA NPs). Intravenous administration in MCAO rat models enhanced the stability of scutellarin, improved its penetration across the BBB, prolonged systemic circulation, and increased its accumulation in the brains of ischemic rats (Yang C. et al., 2022). In summary, nano-carrier technology effectively addresses key delivery challenges, such as BBB penetration and rapid metabolic clearance, faced by SB-derived polyphenolic neuroprotective agents. These systems significantly enhance their bioavailability, promote targeted distribution and retention in the brain, and demonstrate compelling neuroprotective effects across multiple animal models, highlighting a highly promising strategy for central nervous system drug delivery.

## 6 Toxic effects of *Scutellaria baicalensis* polyphenols

While the long-term clinical use of SB in traditional Chinese medicine attests to its relative safety, modern toxicological studies reveal that some of its isolated polyphenolic metabolites can exhibit potential toxicity under specific conditions. These metabolites display a notable dose-dependent bidirectional effect: neuroprotective within the therapeutic window, yet toxic upon supra-threshold exposure (Table 2). For instance, Pudilan anti-inflammatory oral liquid, which contains baicalin as a primary active metabolite, demonstrated dose-dependent developmental toxicity in zebrafish embryos. High-concentration exposure (0.23–0.29 mg/mL) induced significant embryotoxicity, manifested as high mortality, specific malformations (particularly in the tail), and severe inhibition of embryonic viability and development. It also provoked multi-dimensional developmental toxicity, including reduced heart rate, shortened body length, and impaired spontaneous movement (Jingjing et al., 2025). Prenatal exposure to a high dose (90 mg/kg) of baicalein in female mice severely affected fertility, resulting in a reduced number of live fetuses and increased pre- and post-implantation loss (Vaadala et al., 2019). Administration of a high dose of wogonin (40 mg/kg, intravenous injection) induced developmental toxicity in pregnant rats and their fetuses, characterized by significantly suppressed maternal weight gain, increased absorption rate, decreased live fetus rate, and abnormal fetal skeletal development (Zhao et al., 2011). Furthermore, long-term high-dose wogonin (120 mg/kg, intravenous injection) caused cardiac injury in rats, including lesions such as myocardial fibrosis (Qi et al., 2009). The median lethal dose (LD_50_) of chrysin was determined to be 4350 mg/kg. After 90 days of oral administration at 1,000 mg/kg, rats exhibited toxic effects such as abnormal biochemical indices in the liver and kidneys, altered hematological parameters, and histopathological damage (Yao et al., 2021). In summary, baicalin, baicalein, wogonin, and chrysin demonstrate a higher propensity for inducing *in vivo* systemic toxicities, including developmental toxicity and organ damage. In contrast, ferulic acid’s toxicity was observed *in vitro* at a high concentration (40 mg/L), where it suppressed the viability of L929 fibroblasts and hindered wound healing (Truzzi et al., 2020; Talbott et al., 2022). A comparison of toxic dose thresholds reveals that chrysin has the highest *in vivo* toxicity threshold, whereas wogonin possesses the lowest. This indicates that the dose control of wogonin mandates prioritized attention during the clinical translation of SB polyphenols to prevent toxicity resulting from improper dosing. Furthermore, as these polyphenols are often intended for long-term management of chronic conditions, special populations such as pregnant and lactating women, who are more susceptible to drug toxicity, require precise definition of the therapeutic window to ensure clinical safety.

**TABLE 2:** Summary of key toxicological data of polyphenolic metabolites from *Scutellaria baicalensis*.

Active metabolite	Toxic dose	Experimental model	Observed toxicity	References
baicalin	High-concentration exposure (0.23–0.29 mg/mL)	Zebrafish embryos	1. Embryotoxicity: Elevated mortality, tail malformation 2. Developmental toxicity: Decreased heart rate, shortened body length, compromised spontaneous locomotion	Jingjing et al. (2025)
Baicalein	90 mg/kg	adult female Wistar mice	Fertility impairment: Reduced number of live fetuses, increased pre- and post-implantation loss	Vaadala et al. (2019)
wogonin	40 mg/kg	SD rats	Developmental toxicity: Reduced maternal weight gain, increased fetal resorption rate, decreased live fetus rate, fetal skeletal dysplasia 2. Cardiac toxicity: Myocardial fibrosis	Zhao et al. (2011)
Wogonin	120 mg/kg	SD rats	Cardiac toxicity: Myocardial fibrosis	Qi et al. (2009)
Chrysin	1000 mg/kg	SD rats	1. Hepatorenal toxicity: Altered hepatic (ALT, AST, GGT significantly increased) and renal (Cr significantly increased) biochemical parameters 2. Hematological abnormalities: levels of MCH and MCHC were significantly decreased 3. Histopathological damage: Visible lesions in liver and kidney tissues	Yao et al. (2021)
ferulic acid	40 mg/L	L929 mouse fibroblasts	1. Cytotoxicity: Markedly decreased viability of fibroblasts 2. Wound healing inhibition: Suppressed fibroblast migration and wound closure efficiency	Truzzi et al. (2020)

ALT: alanine aminotransferase, AST: aspartate aminotransferase, GGT: gamma glutamyl transferase, Cr: Creatinine, MCH: mean corpuscular hemoglobin, MCHC: mean corpuscular hemoglobin concentration.

## 7 Discussion

Ischemic stroke is characterized by the sudden occlusion of cerebral arteries, leading to a significant reduction in regional cerebral blood flow. This triggers neuronal energy metabolism failure and ultimately results in irreversible neuronal damage. Survivors often experience long-term functional impairments, imposing a substantial economic burden on society (Feng et al., 2025). Although reperfusion therapy remains the cornerstone of ischemic stroke management, its application is limited by a narrow therapeutic time window, the risk of reperfusion injury, and the shortcomings of single-target pharmacological agents. Therefore, there is a pressing need to explore alternative therapeutic strategies. This review provides a comprehensive analysis of the pharmacological effects of major polyphenolic metabolites derived from SB in the treatment of ischemic stroke. The therapeutic potential of these polyphenols is demonstrated through multiple mechanisms, including anti-inflammatory, antioxidant, and anti-apoptotic effects, modulation of neurotransmitters, preservation of BBB integrity, and inhibition of ferroptosis. Notably, baicalein, baicalin, and scutellarin exhibit significant neuroprotective properties, contributing to multi-target synergistic modulation of the pathological cascade in ischemic stroke.

The neuroprotective mechanisms of SB polyphenols are multifaceted. Research indicates that baicalein, baicalin, and scutellarin alleviate neuroinflammation following ischemic stroke. Their action primarily involves targeting and inhibiting the TLR4/NF-κB and MAPK signaling pathways, thereby synergistically promoting microglial polarization toward an anti-inflammatory phenotype, suppressing aberrant activation of astrocytes, facilitating a beneficial shift in the inflammatory microenvironment, and downregulating levels of pro-inflammatory cytokines. Consequently, they effectively inhibit the post-ischemic neuroinflammatory cascade. In terms of antioxidant activity, these polyphenols not only directly reduce levels of ROS and MDA while elevating GSH, but also inhibit 12/15-LOX and NOX to mitigate oxidative stress damage. Furthermore, they activate the Nrf2 pathway and upregulate the expression of downstream antioxidant enzymes, thereby enhancing cellular antioxidant capacity. Regarding anti-apoptotic effects, SB polyphenols counteract the apoptotic cascade through multi-target actions, including activation of the PI3K/Akt survival signaling pathway, upregulation of the anti-apoptotic protein Bcl-2, downregulation of the pro-apoptotic protein Bax, and enhancement of neuronal survival via modulation of the BDNF-TrkB signaling axis. In neurotransmitter regulation, various polyphenolic metabolites from SB modulate glutamate and GABA levels, ameliorating excitotoxicity and exerting neuroprotective effects. Additionally, these metabolites protect BBB integrity, reduce leakage, and alleviate cerebral edema. Notably, baicalein precisely targets multiple key nodes within the ferroptosis pathway in ischemic stroke, achieving synergistic inhibition via a dual mechanism. On one hand, it modulates the balance of the GPX4/ACSL axis by upregulating GPX4 and ACSL3 expression while suppressing ACSL4 activity. On the other hand, it activates the SIRT6-FOXA2-SLC7A11 pathway to enhance GSH synthesis and downregulate ACSL4 expression. Consequently, baicalein blocks the execution of ferroptosis concurrently by facilitating the clearance of lipid peroxides and reducing the production of pro-ferroptotic substrates.

The polyphenolic metabolites from SB demonstrate considerable therapeutic promise for ischemic stroke due to their well-defined neuroprotective activities. However, their clinical translation faces two major obstacles: insufficient bioavailability that hinders achieving effective therapeutic concentrations in the brain, and dose-dependent toxicity that narrows the safe therapeutic window. To address these delivery challenges, various nanodelivery systems have been developed to enhance brain targeting and delivery efficiency through distinct mechanisms. For example, intranasal administration of PEG-PLA micelles and liposomes shortens the drug’s pathway to the brain while reducing systemic metabolism. TPGS-modified liposomes significantly improve oral bioavailability, making them suitable for long-term management of chronic neurological disorders, whereas PLGA nanoparticles enhance the stability of intravenous formulations and promote blood-brain barrier penetration. Collectively, nanocarrier technology provides a crucial foundation for translating the *in vitro* efficacy of these polyphenols into *in vivo* outcomes through optimized delivery routes and functional carrier design. Furthermore, modern toxicological studies reveal that these metabolites exhibit dose-dependent bidirectional effects with significant variations in target organs, toxicity thresholds, and susceptibility across populations. This evidence demands rigorous clinical safety measures, emphasizing the need for precision dosing strategies to prevent acute and chronic toxicity. Special consideration should be given to vulnerable populations, and integrated pharmacokinetic-toxicological studies are essential to establish a safe therapeutic window.

A critical consideration in advancing the clinical translation of SB polyphenols is the strategic integration of delivery efficiency enhancement with toxicity risk management. It is imperative to avoid disproportionately focusing on delivery optimization at the expense of altering toxicity thresholds, or conversely, allowing toxicity concerns to unduly restrict the application of effective delivery strategies. A key step involves systematically evaluating whether nanocarriers, while increasing drug concentrations, cause a shift in the toxicity threshold, thereby preventing the convergence of therapeutic and toxic doses. Furthermore, individualized dosing regimens should be developed based on the distinct pharmacokinetic profiles of various administration routes (oral, intranasal, intravenous). For special populations such as pregnant women or patients with hepatic/renal impairment, population pharmacokinetic studies are needed to establish dose adjustment factors. Long-term medication scenarios necessitate thorough drug accumulation toxicity assessments. Currently, most relevant studies rely on animal models (e.g., mice, rats, zebrafish), whose results have inherent limitations for clinical extrapolation. Consequently, subsequent research should progressively incorporate human pharmacokinetic pilot trials and early-phase clinical safety evaluations to generate more instructive evidence for translation. Therefore, systematically resolving these delivery and toxicity issues is a fundamental prerequisite for transitioning these polyphenols from basic research into safe and effective clinical therapies.

Furthermore, it is crucial to extend focus to the potential PAINS properties of SB polyphenols in in vitro activity screening. Although the studies cited in this review employed multi-tiered experimental designs to verify specificity, the *in vitro* activity evaluation system requires continuous refinement in future research to further minimize interference risks. This can be achieved by employing more target-specific cellular reporter systems, integrating chemical biology probe technologies, or performing structural optimization of lead metabolites to mitigate PAINS liabilities. These strategies will help elucidate the genuine pharmacological mechanisms of these polyphenols, enhance their translational value as ischemic stroke therapeutics, and provide a reliable basis for subsequent candidate drug development.

## 8 Conclusion

The polyphenolic metabolites of SB demonstrate significant potential for ischemic stroke treatment, operating through multi-target and multi-pathway neuroprotective mechanisms. However, their development is hampered by poor bioavailability, rapid metabolism, limited brain distribution, and dose-dependent organotoxicity or developmental toxicity associated with certain metabolites. While nanodelivery systems offer a viable strategy to enhance brain targeting and bioavailability, their clinical translation presents challenges that require systematic evaluation of the safety window, delivery efficiency, and toxicity thresholds. Future research should integrate multi-tiered validation strategies, refine *in vitro* activity assessment models, and advance preclinical and clinical studies. This approach aims to enable their effective integration with existing therapies, potentially providing a novel strategy to delay the progression of neurological deficits following ischemic stroke.

## References

[B1] Akao T. Kawabata K. Yanagisawa E. Ishihara K. Mizuhara Y. Wakui Y. (2000). Baicalin, the predominant flavone glucuronide of scutellariae radix, is absorbed from the rat gastrointestinal tract as the aglycone and restored to its original form. J. Pharm. Pharmacol. 52 (12), 1563–1568. 10.1211/0022357001777621 11197087

[B2] Alsbrook D. L. DI Napoli M. Bhatia K. Biller J. Andalib S. Hinduja A. (2023). Neuroinflammation in acute ischemic and hemorrhagic stroke. Curr. neurology Neurosci. Rep. 23 (8), 407–431. 10.1007/s11910-023-01282-2 37395873 PMC10544736

[B3] An H. M. Li M. N. Yang H. Pang H. Q. Qu C. Xu Y. (2021). A validated UHPLC-MS/MS method for pharmacokinetic and brain distribution studies of twenty constituents in rat after oral administration of jia-wei-qi-fu-yin. J. Pharm. Biomed. analysis 202, 114140. 10.1016/j.jpba.2021.114140 34015592

[B4] Arumugam H. Wong K. H. Low Z. Y. Lal S. Choo W. S. (2025). Plant extracts as a source of antiviral agents against influenza A virus. J. Appl. Microbiol. 136 (3), lxaf056. 10.1093/jambio/lxaf056 40058769

[B5] Bolz S. N. Adasme M. F. Schroeder M. (2021). Toward an understanding of pan-assay interference compounds and promiscuity: a structural perspective on binding modes. J. Chem. Inf. Model. 61 (5), 2248–2262. 10.1021/acs.jcim.0c01227 33899463

[B6] Chen H. Yoshioka H. Kim G. S. Jung J. E. Okami N. Sakata H. (2011). Oxidative stress in ischemic brain damage: mechanisms of cell death and potential molecular targets for neuroprotection. Antioxidants & redox Signal. 14 (8), 1505–1517. 10.1089/ars.2010.3576 20812869 PMC3061196

[B7] Chen H. Guan B. Chen X. Chen X. Li C. Qiu J. (2018). Baicalin attenuates blood-brain barrier disruption and hemorrhagic transformation and improves neurological outcome in ischemic stroke rats with delayed t-PA treatment: involvement of ONOO(-)-MMP-9 pathway. Transl. stroke Res. 9 (5), 515–529. 10.1007/s12975-017-0598-3 29275501

[B8] Cheng C. Y. Ho T. Y. Lee E. J. Su S. Y. Tang N. Y. Hsieh C. L. (2008). Ferulic acid reduces cerebral infarct through its antioxidative and anti-inflammatory effects following transient focal cerebral ischemia in rats. Am. J. Chin. Med. 36 (6), 1105–1119. 10.1142/s0192415x08006570 19051339

[B9] Cheng Z. Tu J. Wang K. Li F. He Y. Wu W. (2024). Wogonin alleviates NLRP3 inflammasome activation after cerebral ischemia-reperfusion injury by regulating AMPK/SIRT1. Brain Res. Bull. 207, 110886. 10.1016/j.brainresbull.2024.110886 38253131

[B10] Ciesielska A. Matyjek M. Kwiatkowska K. (2021). TLR4 and CD14 trafficking and its influence on LPS-induced pro-inflammatory signaling. Cell. Mol. life Sci. CMLS 78 (4), 1233–1261. 10.1007/s00018-020-03656-y 33057840 PMC7904555

[B11] Costine B. Zhang M. Chhajed S. Pearson B. Chen S. Nadakuduti S. S. (2022). Exploring native Scutellaria species provides insight into differential accumulation of flavones with medicinal properties. Sci. Rep. 12 (1), 13201. 10.1038/s41598-022-17586-1 35915209 PMC9343603

[B12] Cui L. Zhang X. Yang R. Liu L. Wang L. Li M. (2010). Baicalein is neuroprotective in rat MCAO model: role of 12/15-lipoxygenase, mitogen-activated protein kinase and cytosolic phospholipase A2. Pharmacol. Biochem. Behav. 96 (4), 469–475. 10.1016/j.pbb.2010.07.007 20637223

[B13] Dang Y. An C. Li Y. Han D. Liu X. Zhang F. (2019). Neutrophil-mediated and low density lipoprotein receptor-mediated dual-targeting nanoformulation enhances brain accumulation of scutellarin and exerts neuroprotective effects against ischemic stroke. RSC Adv. 9 (3), 1299–1318. 10.1039/c8ra06688d 35518053 PMC9059646

[B14] Das S. Mccloskey K. Nepal B. Kortagere S. (2025). EAAT2 activation regulates glutamate excitotoxicity and reduces impulsivity in a rodent model of Parkinson's Disease. Mol. Neurobiol. 62 (5), 5787–5803. 10.1007/s12035-024-04644-0 39630405 PMC11953204

[B15] Deng M. Sun J. Peng L. Huang Y. Jiang W. Wu S. (2022). Scutellarin acts on the AR-NOX axis to remediate oxidative stress injury in a mouse model of cerebral ischemia/reperfusion injury. Phytomedicine Int. J. phytotherapy Phytopharm. 103, 154214. 10.1016/j.phymed.2022.154214 35689902

[B16] Deng X. Chu W. Zhang H. Peng Y. (2023). Nrf2 and ferroptosis: a new research direction for ischemic stroke. Cell. Mol. Neurobiol. 43 (8), 3885–3896. 10.1007/s10571-023-01411-y 37728817 PMC11407729

[B17] Dong D. Quan E. Yuan X. Xie Q. Li Z. Wu B. (2017). Sodium oleate-based nanoemulsion enhances oral absorption of chrysin through inhibition of UGT-Mediated metabolism. Mol. Pharm. 14 (9), 2864–2874. 10.1021/acs.molpharmaceut.6b00851 27983856

[B18] Duan Z. Chen H. Miao W. He J. Xu D. Qi Z. (2024). Scutellarin alleviates microglia-mediated neuroinflammation and apoptosis after ischemic stroke through the PI3K/AKT/GSK3β signaling pathway. J. cell Commun. Signal. 18 (2), e12023. 10.1002/ccs3.12023 38946727 PMC11208122

[B19] Duan Z. Peng Y. Xu D. Yang Y. Wu Y. Wu C. (2025). Scutellarin alleviates neuronal apoptosis in ischemic stroke *via* activation of the PI3K/AKT signaling pathway. Int. J. Mol. Sci. 26 (5), 2175. 10.3390/ijms26052175 40076795 PMC11901123

[B20] Duda-Chodak A. Tarko T. (2023). Possible side effects of polyphenols and their interactions with medicines. Mol. Basel, Switz. 28 (6), 2536. 10.3390/molecules28062536 36985507 PMC10058246

[B21] Dzięcioł M. Wala K. WróBLEWSKA A. Janda-Milczarek K. (2024). The effect of the extraction conditions on the antioxidant activity and bioactive compounds content in ethanolic extracts of Scutellaria baicalensis root. Mol. Basel, Switz. 29 (17), 4153. 10.3390/molecules29174153 39275001 PMC11397618

[B22] EL-Bassossy H. Badawy D. Neamatallah T. Fahmy A. (2016). Ferulic acid, a natural polyphenol, alleviates insulin resistance and hypertension in fructose fed rats: effect on endothelial-dependent relaxation. Chemico-biological Interact. 254, 191–197. 10.1016/j.cbi.2016.06.013 27287418

[B23] Fang C. Liu X. Zhang F. Song T. (2024). Baicalein inhibits cerebral ischemia-reperfusion injury through SIRT6-Mediated FOXA2 deacetylation to promote SLC7A11 expression. eNeuro 11 (10), 0174–24. 10.1523/eneuro.0174-24.2024 39299807 PMC11470267

[B24] Feigin V. L. Owolabi M. O. World Stroke Organization–Lancet Neurology Commission Stroke Collaboration Group (2023). Pragmatic solutions to reduce the global burden of stroke: a World Stroke Organization-Lancet Neurology Commission. Lancet Neurology 22 (12), 1160–1206. 10.1016/s1474-4422(23)00277-6 37827183 PMC10715732

[B25] Feigin V. L. Brainin M. Norrving B. Martins S. O. Pandian J. Lindsay P. (2025). World stroke Organization: global stroke fact sheet 2025. Int. J. stroke official J. Int. Stroke Soc. 20 (2), 132–144. 10.1177/17474930241308142 39635884 PMC11786524

[B26] Feng M. Qin Q. Zhang K. Wang F. Song D. Li M. (2025). Sphk2 in ischemic stroke pathogenesis: roles, mechanisms, and regulation strategies. Ageing Res. Rev. 111, 102844. 10.1016/j.arr.2025.102844 40716518

[B27] Fu L. Cui C. P. Zhang X. Zhang L. (2020). The functions and regulation of Smurfs in cancers. Seminars cancer Biol. 67 (Pt 2), 102–116. 10.1016/j.semcancer.2019.12.023 31899247

[B28] Gan J. Yang X. Wu J. Liu P. Chen Z. Hu Y. (2025). Neuroprotective mechanisms of microglia in ischemic stroke: a review focused on mitochondria. Mol. Biol. Rep. 52 (1), 355. 10.1007/s11033-025-10469-4 40167874

[B29] Ge Y. Wu X. Cai Y. Hu Q. Wang J. Zhang S. (2024). FNDC5 prevents oxidative stress and neuronal apoptosis after traumatic brain injury through SIRT3-dependent regulation of mitochondrial quality control. Cell death & Dis. 15 (5), 364. 10.1038/s41419-024-06748-w 38802337 PMC11130144

[B30] GodíNEZ-Rubí M. Rojas-MayorquíN A. E. OrtuñO-SahagúN D. (2013). Nitric oxide donors as neuroprotective agents after an ischemic stroke-related inflammatory reaction. Oxidative Med. Cell. Longev. 2013, 297357. 10.1155/2013/297357 23691263 PMC3649699

[B31] Gong J. Sun F. Li Y. Zhou X. Duan Z. Duan F. (2015). Momordica charantia polysaccharides could protect against cerebral ischemia/reperfusion injury through inhibiting oxidative stress mediated c-Jun N-terminal kinase 3 signaling pathway. Neuropharmacology 91, 123–134. 10.1016/j.neuropharm.2014.11.020 25510970

[B32] Grabska-Kobyłecka I. Szpakowski P. KróL A. Książek-Winiarek D. Kobyłecki A. Głąbiński A. (2023). Polyphenols and their impact on the prevention of neurodegenerative diseases and development. Nutrients 15 (15), 3454. 10.3390/nu15153454 37571391 PMC10420887

[B33] Greene C. Hanley N. Reschke C. R. Reddy A. Mäe M. A. Connolly R. (2022). Microvascular stabilization *via* blood-brain barrier regulation prevents seizure activity. Nat. Commun. 13 (1), 2003. 10.1038/s41467-022-29657-y 35422069 PMC9010415

[B34] Guo H. Kong S. Chen W. Dai Z. Lin T. Su J. (2014). Apigenin mediated protection of OGD-evoked neuron-like injury in differentiated PC12 cells. Neurochem. Res. 39 (11), 2197–2210. 10.1007/s11064-014-1421-0 25208641

[B35] He Y. Wang R. Mo L. Feng L. (2024). Mediating effects of perceived social support on the relationship between comfort and hope in hospitalized patients with acute ischemic stroke. J. Nurs. Manag. 2024, 6774939. 10.1155/2024/6774939 40224892 PMC11919176

[B36] Herb M. Schramm M. (2021). Functions of ROS in macrophages and antimicrobial immunity. Antioxidants Basel, Switz. 10 (2), 313. 10.3390/antiox10020313 33669824 PMC7923022

[B37] Hernandez K. Jones N. Ortega S. B. (2025). The efficacy of an allosteric modulator of the alpha 7 nicotinic acetylcholine receptor in a murine model of stroke. Front. Neurosci. 19, 1525975. 10.3389/fnins.2025.1525975 40012683 PMC11860958

[B38] Hong Y. Zhao T. Li X. J. Li S. (2016). Mutant huntingtin impairs BDNF release from astrocytes by disrupting conversion of Rab3a-GTP into Rab3a-GDP. J. Neurosci. official J. Soc. Neurosci. 36 (34), 8790–8801. 10.1523/jneurosci.0168-16.2016 27559163 PMC4995297

[B39] Hu Z. Xu D. Meng H. Liu W. Zheng Q. Wang J. (2024). 4-octyl itaconate protects against oxidative stress-induced liver injury by activating the Nrf2/Sirt3 pathway through AKT and ERK1/2 phosphorylation. Biochem. Pharmacol. 220, 115992. 10.1016/j.bcp.2023.115992 38128618

[B40] Huang Z. Guo L. Huang L. Shi Y. Liang J. Zhao L. (2021). Baicalin-loaded macrophage-derived exosomes ameliorate ischemic brain injury *via* the antioxidative pathway. Mater. Sci. & Eng. C, Mater. Biol. Appl. 126, 112123. 10.1016/j.msec.2021.112123 34082940

[B41] Irisa K. Shichita T. (2025). Neural repair mechanisms after ischemic stroke. Inflamm. Regen. 45 (1), 7. 10.1186/s41232-025-00372-7 40098163 PMC11912631

[B42] Jalili S. Panji M. Mahdavimehr M. Mohseni Ahangar A. Shirzad H. Mousavi Nezhad S. A. (2024). Enhancing anti-amyloidogenic properties and antioxidant effects of Scutellaria baicalensis polyphenols through novel nanoparticle formation. Int. J. Biol. Macromol. 262 (Pt 1), 130003. 10.1016/j.ijbiomac.2024.130003 38325696

[B43] Jelinek M. Jurajda M. Duris K. (2021). Oxidative stress in the brain: basic concepts and treatment strategies in stroke. Antioxidants Basel, Switz. 10 (12), 1886. 10.3390/antiox10121886 34942989 PMC8698986

[B44] Jeong S. H. Jang J. H. Cho H. Y. Lee Y. B. (2021). Simultaneous determination of asarinin, β-eudesmol, and wogonin in rats using ultraperformance liquid chromatography-tandem mass spectrometry and its application to pharmacokinetic studies following administration of standards and Gumiganghwal-tang. Biomed. Chromatogr. BMC 35 (4), e5021. 10.1002/bmc.5021 33169364

[B45] Ji Y. Gao Q. Ma Y. Wang F. Tan X. Song D. (2023). An MMP-9 exclusive neutralizing antibody attenuates blood-brain barrier breakdown in mice with stroke and reduces stroke patient-derived MMP-9 activity. Pharmacol. Res. 190, 106720. 10.1016/j.phrs.2023.106720 36893823 PMC11934118

[B46] Jiang P. Gan M. Yen S. H. (2013). Dopamine prevents lipid peroxidation-induced accumulation of toxic α-synuclein oligomers by preserving autophagy-lysosomal function. Front. Cell. Neurosci. 7, 81. 10.3389/fncel.2013.00081 23754979 PMC3668273

[B47] Jiang C. T. Wu W. F. Deng Y. H. Ge J. W. (2020). Modulators of microglia activation and polarization in ischemic stroke (Review). Mol. Med. Rep. 21 (5), 2006–2018. 10.3892/mmr.2020.11003 32323760 PMC7115206

[B48] Jin G. Arai K. Murata Y. Wang S. Stins M. F. Lo E. H. (2008). Protecting against cerebrovascular injury: contributions of 12/15-lipoxygenase to edema formation after transient focal ischemia. Stroke 39 (9), 2538–2543. 10.1161/strokeaha.108.514927 18635843 PMC2754072

[B49] Jingjing C. Lu Z. Yan Z. Li Y. Hou Z. Wang D. (2025). Chinese medicine preparation Pudilan anti-inflammatory oral liquid: chemical constituents and developmental toxicity. J. Ethnopharmacol. 351, 120173. 10.1016/j.jep.2025.120173 40541749

[B50] Kamal F. Z. Lefter R. Jaber H. Balmus I. M. Ciobica A. Iordache A. C. (2023). The role of potential oxidative biomarkers in the prognosis of acute ischemic stroke and the exploration of antioxidants as possible preventive and treatment options. Int. J. Mol. Sci. 24 (7), 6389. 10.3390/ijms24076389 37047362 PMC10094154

[B51] Kang J. B. Park D. J. Shah M. A. Koh P. O. (2022). Quercetin ameliorates glutamate toxicity-induced neuronal cell death by controlling calcium-binding protein parvalbumin. J. veterinary Sci. 23 (2), e26. 10.4142/jvs.21273 35187882 PMC8977545

[B52] Kang C. Li X. Liu P. Liu Y. Niu Y. Zeng X. (2023). Tolerogenic dendritic cells and TLR4/IRAK4/NF-κB signaling pathway in allergic rhinitis. Front. Immunol. 14, 1276512. 10.3389/fimmu.2023.1276512 37915574 PMC10616250

[B53] Kang J. B. Son H. K. Park D. J. Jin Y. B. Shah F. A. Koh P. O. (2024a). Modulation of thioredoxin by chlorogenic acid in an ischemic stroke model and glutamate-exposed neurons. Neurosci. Lett. 825, 137701. 10.1016/j.neulet.2024.137701 38395190

[B54] Kang J. B. Son H. K. Park D. J. Jin Y. B. Koh P. O. (2024b). Chlorogenic acid regulates the expression of protein phosphatase 2A subunit B in the cerebral cortex of a rat stroke model and glutamate-exposed neurons. Laboratory animal Res. 40 (1), 8. 10.1186/s42826-024-00196-5 38429854 PMC10905799

[B55] Kazi M. Alhajri A. Alshehri S. M. Elzayat E. M. Al Meanazel O. T. Shakeel F. (2020). Enhancing oral bioavailability of apigenin using a bioactive self-nanoemulsifying drug delivery System (Bio-SNEDDS): *in vitro,*, *in vivo* and stability evaluations. Pharmaceutics 12 (8), 749. 10.3390/pharmaceutics12080749 32785007 PMC7465069

[B56] Khombi Shooshtari M. Farbood Y. Mansouri S. M. T. Badavi M. Khorsandi L. S. Ghasemi Dehcheshmeh M. (2021). Neuroprotective effects of chrysin mediated by estrogenic receptors following cerebral ischemia and reperfusion in Male rats. Basic Clin. Neurosci. 12 (1), 149–162. 10.32598/bcn.12.1.2354.1 33995936 PMC8114856

[B57] Kim J. Y. Park J. Lee J. E. Yenari M. A. (2017). NOX inhibitors - a promising avenue for ischemic stroke. Exp. Neurobiol. 26 (4), 195–205. 10.5607/en.2017.26.4.195 28912642 PMC5597550

[B58] Kumar G. Mukherjee S. Paliwal P. Singh S. S. Birla H. Singh S. P. (2019). Neuroprotective effect of chlorogenic acid in global cerebral ischemia-reperfusion rat model. Naunyn-Schmiedeberg's archives Pharmacol. 392 (10), 1293–1309. 10.1007/s00210-019-01670-x 31190087

[B59] Lai M. Y. Hsiu S. L. Tsai S. Y. Hou Y. C. Chao P. D. L. (2003). Comparison of metabolic pharmacokinetics of baicalin and baicalein in rats. J. Pharm. Pharmacol. 55 (2), 205–209. 10.1211/002235702522 12631413

[B60] Lai T. W. Zhang S. Wang Y. T. (2014). Excitotoxicity and stroke: identifying novel targets for neuroprotection. Prog. Neurobiol. 115, 157–188. 10.1016/j.pneurobio.2013.11.006 24361499

[B61] Lapchak P. A. Maher P. Schubert D. Zivin J. A. (2007). Baicalein, an antioxidant 12/15-lipoxygenase inhibitor improves clinical rating scores following multiple infarct embolic strokes. Neuroscience 150 (3), 585–591. 10.1016/j.neuroscience.2007.09.033 17942241

[B62] Lee K. H. Cha M. Lee B. H. (2020). Neuroprotective effect of antioxidants in the brain. Int. J. Mol. Sci. 21 (19), 7152. 10.3390/ijms21197152 32998277 PMC7582347

[B63] Li H. Y. Yuan Z. Y. Wang Y. G. Wan H. J. Hu J. Chai Y. S. (2012). Role of baicalin in regulating toll-like receptor 2/4 after ischemic neuronal injury. Chin. Med. J. 125 (9), 1586–1593. 10.3760/cma.j.issn.0366-6999.2012.09.012 22800826

[B64] Li X. Xu M. Ding L. Tang J. (2019). MiR-27a: a novel biomarker and potential therapeutic target in tumors. J. Cancer 10 (12), 2836–2848. 10.7150/jca.31361 31258791 PMC6584939

[B65] Li C. Sui C. Wang W. Yan J. Deng N. Du X. (2021a). Baicalin Attenuates oxygen-glucose Deprivation/Reoxygenation-Induced injury by modulating the BDNF-TrkB/PI3K/Akt and MAPK/Erk1/2 signaling axes in Neuron-Astrocyte cocultures. Front. Pharmacol. 12, 599543. 10.3389/fphar.2021.599543 34234667 PMC8255628

[B66] Li L. Sluter M. N. Yu Y. Jiang J. (2021b). Prostaglandin E receptors as targets for ischemic stroke: novel evidence and molecular mechanisms of efficacy. Pharmacol. Res. 163, 105238. 10.1016/j.phrs.2020.105238 33053444 PMC7854947

[B67] Li S. He Y. Chen K. Sun J. Zhang L. He Y. (2021c). RSL3 drives ferroptosis through NF-κB pathway activation and GPX4 depletion in glioblastoma. Oxidative Med. Cell. Longev. 2021, 2915019. 10.1155/2021/2915019 34987700 PMC8720588

[B68] Li N. Liu Y. Li J. R. Zhang W. X. (2022a). Chrysin, which targets PLAU, protects PC12 cells from OGD/R-stimulated damage through repressing the NF-κB signaling pathway. Regen. Ther. 19, 69–76. 10.1016/j.reth.2021.11.002 35097165 PMC8761957

[B69] Li T. Xu T. Zhao J. Gao H. Xie W. (2022b). Depletion of iNOS-positive inflammatory cells decelerates neuronal degeneration and alleviates cerebral ischemic damage by suppressing the inflammatory response. Free Radic. Biol. & Med. 181, 209–220. 10.1016/j.freeradbiomed.2022.02.008 35150825

[B70] Li M. Meng Z. Yu S. Li J. Wang Y. Yang W. (2022c). Baicalein ameliorates cerebral ischemia-reperfusion injury by inhibiting ferroptosis *via* regulating GPX4/ACSL4/ACSL3 axis. Chemico-biological Interact. 366, 110137. 10.1016/j.cbi.2022.110137 36055377

[B71] Li L. Song J. J. Zhang M. X. Zhang H. W. Zhu H. Y. Guo W. (2023a). Oridonin ameliorates caspase-9-mediated brain neuronal apoptosis in mouse with ischemic stroke by inhibiting RIPK3-mediated mitophagy. Acta Pharmacol. Sin. 44 (4), 726–740. 10.1038/s41401-022-00995-3 36216897 PMC10042824

[B72] Li Y. Liu B. Zhao T. Quan X. Han Y. Cheng Y. (2023b). Comparative study of extracellular vesicles derived from mesenchymal stem cells and brain endothelial cells attenuating blood-brain barrier permeability *via* regulating Caveolin-1-dependent ZO-1 and Claudin-5 endocytosis in acute ischemic stroke. J. nanobiotechnology 21 (1), 70. 10.1186/s12951-023-01828-z 36855156 PMC9976550

[B73] Li J. Ning Z. Zhong X. Hu D. Wang Y. Cheng X. (2024). Dynamic changes in Beclin-1, LC3B, and p62 in aldose reductase-knockout mice at different time points after ischemic stroke. Heliyon 10 (19), e38068. 10.1016/j.heliyon.2024.e38068 39386838 PMC11462252

[B74] Li D. Huo X. Shen L. Qian M. Wang J. Mao S. (2025a). Astrocyte heterogeneity in ischemic stroke: molecular mechanisms and therapeutic targets. Neurobiol. Dis. 209, 106885. 10.1016/j.nbd.2025.106885 40139279

[B75] Li J. Xu Q. Xu X. He W. Zhang H. Ren H. (2025b). Apigenin protects ischemic stroke by regulating intestinal microbiota homeostasis, regulates brain metabolic profile. Front. Pharmacol. 16, 1553081. 10.3389/fphar.2025.1553081 40124778 PMC11925864

[B76] Li Y. F. Zhang Y. F. Huang C. Jiang J. M. (2025c). Baicalin improves neurological outcomes in mice with ischemic stroke by inhibiting astrocyte activation and neuroinflammation. Int. Immunopharmacol. 149, 114186. 10.1016/j.intimp.2025.114186 39923584

[B77] Liang W. Huang X. Chen W. (2017). The effects of baicalin and Baicalein on cerebral ischemia: a review. Aging Dis. 8 (6), 850–867. 10.14336/ad.2017.0829 29344420 PMC5758355

[B78] Liang Z. Lou Y. Hao Y. Li H. Feng J. Liu S. (2023). The relationship of astrocytes and microglia with different stages of ischemic stroke. Curr. Neuropharmacol. 21 (12), 2465–2480. 10.2174/1570159x21666230718104634 37464832 PMC10616922

[B79] Lin B. (2011). Polyphenols and neuroprotection against ischemia and neurodegeneration. Mini Rev. Med. Chem. 11 (14), 1222–1238. 10.2174/13895575111091222 22070681

[B80] Liu H. Li W. Ahmad M. Miller T. M. Rose M. E. Poloyac S. M. (2011). Modification of ubiquitin-C-terminal hydrolase-L1 by cyclopentenone prostaglandins exacerbates hypoxic injury. Neurobiol. Dis. 41 (2), 318–328. 10.1016/j.nbd.2010.09.020 20933087 PMC3014436

[B81] Liu M. Xu Z. Wang L. Zhang L. Liu Y. Cao J. (2020a). Cottonseed oil alleviates ischemic stroke injury by inhibiting the inflammatory activation of microglia and astrocyte. J. neuroinflammation 17 (1), 270. 10.1186/s12974-020-01946-7 32917229 PMC7488511

[B82] Liu W. Wang X. O'Connor M. Wang G. Han F. (2020b). Brain-Derived neurotrophic factor and its potential therapeutic role in stroke comorbidities. Neural plast. 2020, 1969482. 10.1155/2020/1969482 32399020 PMC7204205

[B83] Liu G. Liang Y. Xu M. Sun M. Sun W. Zhou Y. (2021a). Protective mechanism of erigeron breviscapus injection on blood-brain barrier injury induced by cerebral ischemia in rats. Sci. Rep. 11 (1), 18451. 10.1038/s41598-021-97908-x 34531475 PMC8446017

[B84] Liu M. Li H. Zhang L. Xu Z. Song Y. Wang X. (2021b). Cottonseed oil alleviates ischemic stroke-induced oxidative stress injury *via* activating the Nrf2 signaling pathway. Mol. Neurobiol. 58 (6), 2494–2507. 10.1007/s12035-020-02256-y 33443681

[B85] Liu H. Chen X. Liu Y. Fang C. Chen S. (2021c). Antithrombotic effects of huanglian jiedu decoction in a rat model of ischaemia-reperfusion-induced cerebral stroke. Pharm. Biol. 59 (1), 823–827. 10.1080/13880209.2021.1942505 34196572 PMC8253176

[B86] Liu X. Xiao X. Han X. Yao L. Lan W. (2022a). A new therapeutic trend: natural medicine for ameliorating ischemic stroke *via* PI3K/Akt signaling pathway. Mol. Basel, Switz. 27 (22), 7963. 10.3390/molecules27227963 36432062 PMC9694461

[B87] Liu B. H. Pu J. Li Z. Q. Zhang X. R. (2022b). The effects of hypothermia on glutamate and γ-aminobutyric acid metabolism during ischemia in monkeys: a repeated-measures ANOVA study. Sci. Rep. 12 (1), 14470. 10.1038/s41598-022-18783-8 36008544 PMC9411555

[B88] Liu X. Ren X. Li R. Deng Q. Li X. He Y. (2024a). Integrated pharmacokinetic-pharmacodynamic modeling and metabolomic research on polyphenol-rich fraction of Thymus quinquecostatus Celak. Alleviating cerebral ischemia-reperfusion injury. J. Ethnopharmacol. 330, 118229. 10.1016/j.jep.2024.118229 38670403

[B89] Liu R. Yu Y. Ge Q. Feng R. Zhong G. Luo L. (2024b). Genistein-3'-sodium sulfonate promotes brain functional rehabilitation in ischemic stroke rats by regulating astrocytes polarization through NF-κB signaling pathway. Chemico-biological Interact. 400, 111159. 10.1016/j.cbi.2024.111159 39059603

[B90] Liu J. Tang D. Kang R. (2024c). Targeting GPX4 in ferroptosis and cancer: chemical strategies and challenges. Trends Pharmacol. Sci. 45 (8), 666–670. 10.1016/j.tips.2024.05.006 38866667

[B91] Liu Y. Hong J. Wang G. Mei Z. (2025a). An emerging role of SNAREs in ischemic stroke: from pre-to post-diseases. Biochem. Pharmacol. 236, 116907. 10.1016/j.bcp.2025.116907 40158821

[B92] Liu T. Li X. Zhou X. Chen W. Wen A. Liu M. (2025b). PI3K/AKT signaling and neuroprotection in ischemic stroke: molecular mechanisms and therapeutic perspectives. Neural Regen. Res. 20 (10), 2758–2775. 10.4103/nrr.Nrr-d-24-00568 39435629 PMC11826468

[B93] Lu Y. Joerger R. Wu C. (2011). Study of the chemical composition and antimicrobial activities of ethanolic extracts from roots of Scutellaria baicalensis Georgi. J. Agric. food Chem. 59 (20), 10934–10942. 10.1021/jf202741x 21866919

[B94] Luo X. Chen X. Shen X. Yang Z. Du G. (2019). Rapid identification and analysis of the active components of traditional Chinese medicine xiaoxuming decoction for ischemic stroke treatment by integrating UPLC-Q-TOF/MS and RRLC-QTRAP MS(n) method. J. Chromatogr. B, Anal. Technol. Biomed. life Sci. 1124, 313–322. 10.1016/j.jchromb.2019.06.023 31269467

[B95] Ma W. Liu T. Ogaji O. D. Du K. Chang Y. (2024). Recent advances in Scutellariae radix: a comprehensive review on ethnobotanical uses, processing, phytochemistry, pharmacological effects, quality control and influence factors of biosynthesis. Heliyon 10 (16), e36146. 10.1016/j.heliyon.2024.e36146 39262990 PMC11388511

[B96] MagalhãES P. R. Reis P. Vila-ViçOSA D. Machuqueiro M. Victor B. L. (2021). Identification of pan-assay INterference compoundS (PAINS) using an MD-Based protocol. Methods Mol. Biol. Clift. NJ 2315, 263–271. 10.1007/978-1-0716-1468-6_15 34302681

[B97] Nimgampalle M. Chakravarthy H. Sharma S. Shree S. Bhat A. R. Pradeepkiran J. A. (2023). Neurotransmitter systems in the etiology of major neurological disorders: emerging insights and therapeutic implications. Ageing Res. Rev. 89, 101994. 10.1016/j.arr.2023.101994 37385351

[B98] Ohmori I. Ouchida M. Imai H. Ishida S. Toyokuni S. Mashimo T. (2022). Thioredoxin deficiency increases oxidative stress and causes bilateral symmetrical degeneration in rat midbrain. Neurobiol. Dis. 175, 105921. 10.1016/j.nbd.2022.105921 36372289

[B99] Pandareesh M. D. Mythri R. B. Srinivas Bharath M. M. (2015). Bioavailability of dietary polyphenols: factors contributing to their clinical application in CNS diseases. Neurochem. Int. 89, 198–208. 10.1016/j.neuint.2015.07.003 26163045

[B100] Pang H. Q. Guo J. X. Yang Y. Xu L. Wang J. Yang F. (2024). Elucidating the chemical interaction effects of herb pair Danshen-Chuanxiong and its anti-ischemic stroke activities evaluation. J. Ethnopharmacol. 318 (Pt B), 117058. 10.1016/j.jep.2023.117058 37597675

[B101] Paul S. Candelario-Jalil E. (2021). Emerging neuroprotective strategies for the treatment of ischemic stroke: an overview of clinical and preclinical studies. Exp. Neurol. 335, 113518. 10.1016/j.expneurol.2020.113518 33144066 PMC7869696

[B102] Pawluk H. Tafelska-Kaczmarek A. Sopońska M. Porzych M. Modrzejewska M. Pawluk M. (2024). The influence of oxidative stress markers in patients with ischemic stroke. Biomolecules 14 (9), 1130. 10.3390/biom14091130 39334896 PMC11430825

[B103] Peng X. Mei Z. Luo Z. Ge J. (2025). Stroke with white matter lesions: potential pathophysiology and therapeutic targets. Br. J. Hosp. Med. Lond. Engl. 86 (3), 1–21. 10.12968/hmed.2024.0771 40135304

[B104] Qi Q. Peng J. Liu W. You Q. Yang Y. Lu N. (2009). Toxicological studies of wogonin in experimental animals. Phytotherapy Res. PTR 23 (3), 417–422. 10.1002/ptr.2645 19003942

[B105] Qiu Y. M. Zhang C. L. Chen A. Q. Wang H. L. Zhou Y. F. Li Y. N. (2021). Immune cells in the BBB disruption after Acute ischemic stroke: targets for immune therapy? Front. Immunol. 12, 678744. 10.3389/fimmu.2021.678744 34248961 PMC8260997

[B106] Rahmani A. H. Almatroudi A. Khan A. A. Babiker A. Y. Alanezi M. Allemailem K. S. (2022). The multifaceted role of Baicalein in cancer management through modulation of cell signalling pathways. Mol. Basel, Switz. 27 (22), 8023. 10.3390/molecules27228023 36432119 PMC9692503

[B107] Ran Y. Qie S. Gao F. Ding Z. Yang S. Tian G. (2021). Baicalein ameliorates ischemic brain damage through suppressing proinflammatory microglia polarization *via* inhibiting the TLR4/NF-κB and STAT1 pathway. Brain Res. 1770, 147626. 10.1016/j.brainres.2021.147626 34418356

[B108] Rodrigues T. Dias A. L. Dos Santos A. M. F. Monteiro A. F. M. Oliveira M. C. N. Pires H. F. O. (2024). Multi-target phenylpropanoids against epilepsy. Curr. Neuropharmacol. 22 (13), 2168–2190. 10.2174/1570159x22666240524160126 38847378 PMC11337686

[B109] Salimi R. Naderi R. Shirpoor A. (2023). Involvement of miR-27a/smurf1/TNF-α and mitochondrial apoptotic pathway in apoptosis induced by cerebral ischemia-reperfusion injury in rats: the protective effect of chlorogenic acid. Neurosci. Lett. 817, 137529. 10.1016/j.neulet.2023.137529 37871828

[B110] Sarkaki A. Farbood Y. Mansouri S. M. T. Badavi M. Khorsandi L. Dehcheshmeh M. G. (2019). Chrysin prevents cognitive and hippocampal long-term potentiation deficits and inflammation in rat with cerebral hypoperfusion and reperfusion injury. Life Sci. 226, 202–209. 10.1016/j.lfs.2019.04.027 30991061

[B111] Shah M. A. Kang J. B. Park D. J. Kim M. O. Koh P. O. (2022a). Chlorogenic acid alleviates cerebral ischemia-induced neuroinflammation *via* attenuating nuclear factor kappa B activation. Neurosci. Lett. 773, 136495. 10.1016/j.neulet.2022.136495 35108588

[B112] Shah M. A. Kang J. B. Koh P. O. (2022b). Chlorogenic acid modulates the ubiquitin-proteasome system in stroke animal model. Laboratory animal Res. 38 (1), 41. 10.1186/s42826-022-00151-2 36539905 PMC9768937

[B113] Shah M. A. Kang J. B. Kim M. O. Koh P. O. (2022c). Chlorogenic acid alleviates the reduction of Akt and bad phosphorylation and of phospho-bad and 14-3-3 binding in an animal model of stroke. J. veterinary Sci. 23 (6), e84. 10.4142/jvs.22200 36259103 PMC9715392

[B114] Shah M. A. Kang J. B. Koh P. O. (2023). Identification of proteins regulated by chlorogenic acid in an ischemic animal model: a proteomic approach. Laboratory animal Res. 39 (1), 12. 10.1186/s42826-023-00164-5 37271817 PMC10240784

[B115] Song X. Gong Z. Liu K. Kou J. Liu B. (2020). Baicalin combats glutamate excitotoxicity *via* protecting glutamine synthetase from ROS-induced 20S proteasomal degradation. Redox Biol. 34, 101559. 10.1016/j.redox.2020.101559 32473460 PMC7260594

[B116] Stragierowicz J. Daragó A. Brzeźnicki S. Kilanowicz A. (2017). Optimization of ultra-performance liquid chromatography (UPLC) with fluorescence detector (FLD) method for the quantitative determination of selected neurotransmitters in rat brain. Med. Pr. 68 (5), 583–591. 10.13075/mp.5893.00622 28749489

[B117] Sun Y. Zhao Y. Yao J. Zhao L. Wu Z. Wang Y. (2015). Wogonoside protects against dextran sulfate sodium-induced experimental colitis in mice by inhibiting NF-κB and NLRP3 inflammasome activation. Biochem. Pharmacol. 94 (2), 142–154. 10.1016/j.bcp.2015.02.002 25677765

[B118] Takahashi H. Chen M. C. Pham H. Angst E. King J. C. Park J. (2011). Baicalein, a component of Scutellaria baicalensis, induces apoptosis by Mcl-1 down-regulation in human pancreatic cancer cells. Biochimica biophysica acta 1813 (8), 1465–1474. 10.1016/j.bbamcr.2011.05.003 21596068 PMC3123440

[B119] Talbi A. Zhao D. Liu Q. Li J. Fan A. Yang W. (2014). Pharmacokinetics, tissue distribution, excretion and plasma protein binding studies of wogonin in rats. Mol. Basel, Switz. 19 (5), 5538–5549. 10.3390/molecules19055538 24786691 PMC6270787

[B120] Talbott H. E. Mascharak S. Griffin M. Wan D. C. Longaker M. T. (2022). Wound healing, fibroblast heterogeneity, and fibrosis. Cell stem cell 29 (8), 1161–1180. 10.1016/j.stem.2022.07.006 35931028 PMC9357250

[B121] Teleanu R. I. Niculescu A. G. Roza E. Vladâcenco O. Grumezescu A. M. Teleanu D. M. (2022). Neurotransmitters-Key factors in neurological and neurodegenerative disorders of the central nervous system. Int. J. Mol. Sci. 23 (11), 5954. 10.3390/ijms23115954 35682631 PMC9180936

[B122] Tian X. Li X. Pan M. Yang L. Z. Li Y. Fang W. (2024). Progress of ferroptosis in ischemic stroke and therapeutic targets. Cell. Mol. Neurobiol. 44 (1), 25. 10.1007/s10571-024-01457-6 38393376 PMC10891262

[B123] Tong M. Wu X. Zhang S. Hua D. Li S. Yu X. (2022). Application of TPGS as an efflux inhibitor and a plasticizer in baicalein solid dispersion. Eur. J. Pharm. Sci. official J. Eur. Fed. Pharm. Sci. 168, 106071. 10.1016/j.ejps.2021.106071 34774716

[B124] Trofin D. M. Sardaru D. P. Trofin D. Onu I. Tutu A. Onu A. (2025). Oxidative stress in brain function. Antioxidants Basel, Switz. 14 (3), 297. 10.3390/antiox14030297 40227270 PMC11939459

[B125] Truzzi F. Valerii M. C. Tibaldi C. Zhang Y. Abduazizova V. Spisni E. (2020). Are supplements safe? Effects of gallic and ferulic acids on *in vitro* cell models. Nutrients 12 (6), 1591. 10.3390/nu12061591 32485864 PMC7352663

[B126] Tsai T. H. Liu S. C. Tsai P. L. Ho L. K. Shum A. Y. C. Chen C. F. (2002). The effects of the cyclosporin A, a P-glycoprotein inhibitor, on the pharmacokinetics of baicalein in the rat: a microdialysis study. Br. J. Pharmacol. 137 (8), 1314–1320. 10.1038/sj.bjp.0704959 12466241 PMC1573598

[B127] Vaadala S. Ponneri N. Karnam V. S. Pamuru R. R. (2019). Baicalein, a flavonoid, causes prolonged estrus and suppressed fertility output upon prenatal exposure in female mice. Iran. J. basic Med. Sci. 22 (4), 452–459. 10.22038/ijbms.2019.33376.7972 31168352 PMC6535191

[B128] VAN Leyen K. Kim H. Y. Lee S. R. Jin G. Arai K. Lo E. H. (2006). Baicalein and 12/15-lipoxygenase in the ischemic brain. Stroke 37 (12), 3014–3018. 10.1161/01.STR.0000249004.25444.a5 17053180

[B129] Waheed A. Zameer S. Ashrafi K. Ali A. Sultana N. Aqil M. (2023). Insights into pharmacological potential of apigenin through various pathways on a nanoplatform in multitude of diseases. Curr. Pharm. Des. 29 (17), 1326–1340. 10.2174/1381612829666230529164321 37254541

[B130] Wan F. Wang M. Zhong R. Chen L. Han H. Liu L. (2021). Supplementation with Chinese medicinal plant extracts from Lonicera hypoglauca and Scutellaria baicalensis mitigates colonic inflammation by regulating oxidative stress and gut microbiota in a colitis mouse model. Front. Cell. Infect. Microbiol. 11, 798052. 10.3389/fcimb.2021.798052 35059326 PMC8763710

[B131] Wang L. Zhang D. Wang N. Li S. Tan H. Y. Feng Y. (2019). Polyphenols of Chinese skullcap roots: from chemical profiles to anticancer effects. RSC Adv. 9 (44), 25518–25532. 10.1039/c9ra03229k 35530094 PMC9070317

[B132] Wang X. Zhang C. Han N. Luo J. Zhang S. Wang C. (2021a). Triglyceride-mimetic prodrugs of scutellarin enhance oral bioavailability by promoting intestinal lymphatic transport and avoiding first-pass metabolism. Drug Deliv. 28 (1), 1664–1672. 10.1080/10717544.2021.1960928 34338567 PMC8330727

[B133] Wang L. Chen Y. Feng D. Wang X. (2021b). Serum ICAM-1 as a predictor of prognosis in patients with acute ischemic stroke. BioMed Res. Int. 2021, 5539304. 10.1155/2021/5539304 33791362 PMC7997739

[B134] Wang C. Liu Y. Liu X. Zhang Y. Yan X. Deng X. (2023). Scutellarin alleviates ischemic brain injury in the acute phase by affecting the activity of neurotransmitters in neurons. Mol. Basel, Switz. 28 (7), 3181. 10.3390/molecules28073181 37049959 PMC10095904

[B135] Wang H. Ma J. Li X. Peng Y. Wang M. (2024). FDA compound library screening Baicalin upregulates TREM2 for the treatment of cerebral ischemia-reperfusion injury. Eur. J. Pharmacol. 969, 176427. 10.1016/j.ejphar.2024.176427 38428662

[B136] Wang X. Shao Q. Gao Y. (2025). The emerging role of 12/15-lipoxygenase in ischemic stroke. Brain Res. Bull. 221, 111194. 10.1016/j.brainresbull.2025.111194 39788462

[B137] Xu M. Chen X. Gu Y. Peng T. Yang D. Chang R. C. C. (2013). Baicalin can scavenge peroxynitrite and ameliorate endogenous peroxynitrite-mediated neurotoxicity in cerebral ischemia-reperfusion injury. J. Ethnopharmacol. 150 (1), 116–124. 10.1016/j.jep.2013.08.020 23973788

[B138] Xu D. Kong T. Shao Z. Liu M. Zhang R. Zhang S. (2021a). Orexin-A alleviates astrocytic apoptosis and inflammation *via* inhibiting OX1R-mediated NF-κB and MAPK signaling pathways in cerebral ischemia/reperfusion injury. Biochimica biophysica acta Mol. basis Dis. 1867 (11), 166230. 10.1016/j.bbadis.2021.166230 34358627

[B139] Xu Y. Liu Y. Li K. Miao S. Lv C. Wang C. (2021b). Regulation of PGE(2) pathway during cerebral ischemia reperfusion injury in rat. Cell. Mol. Neurobiol. 41 (7), 1483–1496. 10.1007/s10571-020-00911-5 32621176 PMC11448554

[B140] Xu D. Zhang L. Meng H. Zhao W. Hu Z. Wang J. (2024a). Exploring the anti-ischemic stroke potential of wogonoside: insights from Nrf2/Sirt3 signaling pathway and UPLC-TripleTOF-MS/MS-based metabolomics. J. Pharm. Biomed. analysis 246, 116206. 10.1016/j.jpba.2024.116206 38733762

[B141] Xu S. Wang D. Tan L. Lu J. (2024b). The role of NLRP3 inflammasome in type 2 inflammation related diseases. Autoimmunity 57 (1), 2310269. 10.1080/08916934.2024.2310269 38332696

[B142] Xu Z. Li Y. Pi P. Yi Y. Tang H. Zhang Z. (2024c). B. glomerulata promotes neuroprotection against ischemic stroke by inhibiting apoptosis through the activation of PI3K/AKT/mTOR pathway. Phytomedicine Int. J. phytotherapy Phytopharm. 132, 155817. 10.1016/j.phymed.2024.155817 39029135

[B143] Yang S. Lian G. (2020). ROS and diseases: role in metabolism and energy supply. Mol. Cell. Biochem. 467 (1-2), 1–12. 10.1007/s11010-019-03667-9 31813106 PMC7089381

[B144] Yang S. Wang H. Yang Y. Wang R. Wang Y. Wu C. (2019). Baicalein administered in the subacute phase ameliorates ischemia-reperfusion-induced brain injury by reducing neuroinflammation and neuronal damage. Biomed. & Pharmacother. = Biomedecine & Pharmacother. 117, 109102. 10.1016/j.biopha.2019.109102 31228802

[B145] Yang B. Dong Y. Wang F. Zhang Y. (2020). Nanoformulations to enhance the bioavailability and physiological functions of polyphenols. Mol. Basel, Switz. 25 (20), 4613. 10.3390/molecules25204613 33050462 PMC7587200

[B146] Yang C. Gong S. Chen X. Wang M. Zhang L. Zhang L. (2021). Analgecine regulates microglia polarization in ischemic stroke by inhibiting NF-κB through the TLR4 MyD88 pathway. Int. Immunopharmacol. 99, 107930. 10.1016/j.intimp.2021.107930 34229178

[B147] Yang Y. Zhu J. Yao C. L. Guo D. A. He N. Mei Q. X. (2022a). Determination of six core components from Mahuang Xuanfei Zhike syrup in rat plasma and tissues by UPLC-MS/MS: application to a pharmacokinetics and tissue distribution study. Biomed. Chromatogr. BMC 36 (12), e5496. 10.1002/bmc.5496 36047933

[B148] Yang C. Zhao Q. Yang S. Wang L. Xu X. Li L. (2022b). Intravenous administration of Scutellarin nanoparticles augments the protective effect against cerebral ischemia-reperfusion injury in rats. Mol. Pharm. 19 (5), 1410–1421. 10.1021/acs.molpharmaceut.1c00942 35441510 PMC9066406

[B149] Yang Y. Zhu T. Wang X. Xiong F. Hu Z. Qiao X. (2022c). ACSL3 and ACSL4, distinct roles in ferroptosis and cancers. Cancers 14 (23), 5896. 10.3390/cancers14235896 36497375 PMC9739553

[B150] Yang R. Wang R. Xu A. Zhang J. Ma J. (2024a). Mitigating neurodegenerative diseases: the protective influence of baicalin and baicalein through neuroinflammation regulation. Front. Pharmacol. 15, 1425731. 10.3389/fphar.2024.1425731 39687298 PMC11647303

[B151] Yang Y. Sun L. Liu X. Liu W. Zhang Z. Zhou X. (2024b). Neurotransmitters: impressive regulators of tumor progression. Biomed. & Pharmacother. = Biomedecine & Pharmacother. 176, 116844. 10.1016/j.biopha.2024.116844 38823279

[B152] Yao W. Cheng J. Kandhare A. D. Mukherjee-Kandhare A. A. Bodhankar S. L. Lu G. (2021). Toxicological evaluation of a flavonoid, chrysin: morphological, behavioral, biochemical and histopathological assessments in rats. Drug Chem. Toxicol. 44 (6), 601–612. 10.1080/01480545.2019.1687510 31724432

[B153] Ye J. Huang F. Zeng H. Xu X. Wu G. Tian S. (2023). Multi-omics and network pharmacology study reveals the effects of Dengzhan Shengmai capsule against neuroinflammatory injury and thrombosis induced by ischemic stroke. J. Ethnopharmacol. 305, 116092. 10.1016/j.jep.2022.116092 36587875

[B154] Yu Z. Su G. Zhang L. Liu G. Zhou Y. Fang S. (2022). Icaritin inhibits neuroinflammation in a rat cerebral ischemia model by regulating microglial polarization through the GPER-ERK-NF-κB signaling pathway. Mol. Med. Camb. Mass 28 (1), 142. 10.1186/s10020-022-00573-7 36447154 PMC9706854

[B155] Yu S. Li D. Shi A. Long Y. Deng J. Ma Y. (2023). Multidrug-loaded liposomes prevent ischemic stroke through intranasal administration. Biomed. & Pharmacother. = Biomedecine & Pharmacother. 162, 114542. 10.1016/j.biopha.2023.114542 36989725

[B156] Yu M. Liu G. Chen W. Qiu Y. You N. Chen S. (2025a). Choline metabolism in ischemic stroke: an underappreciated two-edged sword. Pharmacol. Res. 214, 107685. 10.1016/j.phrs.2025.107685 40054542

[B157] Yu Y. Wang T. Li Q. Zhao H. Li B. Lei D. (2025b). DL-3-n-butylphthalide inhibits astrocyte activation in the cortical penumbra of ischemia-reperfusion model rats *via* AKT signaling. Brain Res. Bull. 225, 111332. 10.1016/j.brainresbull.2025.111332 40185418

[B158] Yuan Y. Rangarajan P. Kan E. M. Wu Y. Wu C. Ling E. A. (2015). Scutellarin regulates the Notch pathway and affects the migration and morphological transformation of activated microglia in experimentally induced cerebral ischemia in rats and in activated BV-2 microglia. J. neuroinflammation 12, 11. 10.1186/s12974-014-0226-z 25600517 PMC4316603

[B159] Zhang J. Cai W. Zhou Y. Liu Y. Wu X. Li Y. (2015). Profiling and identification of the metabolites of baicalin and study on their tissue distribution in rats by ultra-high-performance liquid chromatography with linear ion trap-orbitrap mass spectrometer. J. Chromatogr. B, Anal. Technol. Biomed. life Sci. 985, 91–102. 10.1016/j.jchromb.2015.01.018 25661005

[B160] Zhang X. Fei X. Tao W. Li J. Shen H. Wang X. (2017a). Neuroprotective effect of modified Xijiao Dihuang Decoction against oxygen-glucose deprivation and reoxygenation-induced injury in PC12 cells: involvement of TLR4-MyD88/NF-κB signaling pathway. Evidence-based complementary Altern. Med. eCAM 2017, 3848595. 10.1155/2017/3848595 29234386 PMC5682898

[B161] Zhang Q. Fu X. Wang J. Yang M. Kong L. (2017b). Treatment effects of ischemic stroke by berberine, Baicalin, and Jasminoidin from huang-lian-jie-du-decoction (HLJDD) explored by an integrated metabolomics approach. Oxidative Med. Cell. Longev. 2017, 9848594. 10.1155/2017/9848594 28894512 PMC5574319

[B162] Zhang W. Wei R. Zhang L. Tan Y. Qian C. (2017c). Sirtuin 6 protects the brain from cerebral ischemia/reperfusion injury through NRF2 activation. Neuroscience 366, 95–104. 10.1016/j.neuroscience.2017.09.035 28951325

[B163] Zhang Y. Ouyang L. Mai X. Wang H. Liu S. Zeng H. (2019). Use of UHPLC-QTOF-MS/MS with combination of *in silico* approach for distributions and metabolites profile of flavonoids after oral administration of Niuhuang Shangqing tablets in rats. J. Chromatogr. B, Anal. Technol. Biomed. life Sci. 1114-1115, 55–70. 10.1016/j.jchromb.2019.03.021 30928832

[B164] Zhang P. Yang M. Chen C. Liu L. Wei X. Zeng S. (2020a). Toll-like receptor 4 (TLR4)/Opioid receptor pathway crosstalk and impact on opioid analgesia, immune function, and gastrointestinal motility. Front. Immunol. 11, 1455. 10.3389/fimmu.2020.01455 32733481 PMC7360813

[B165] Zhang L. Yang S. Huang L. Ho P. C. L. (2020b). Poly (ethylene glycol)-block-poly (D, L-lactide) (PEG-PLA) micelles for brain delivery of baicalein through nasal route for potential treatment of neurodegenerative diseases due to oxidative stress and inflammation: an *in vitro* and *in vivo* study. Int. J. Pharm. 591, 119981. 10.1016/j.ijpharm.2020.119981 33069896

[B166] Zhang Y. Swanda R. V. Nie L. Liu X. Wang C. Lee H. (2021). mTORC1 couples cyst(e)ine availability with GPX4 protein synthesis and ferroptosis regulation. Nat. Commun. 12 (1), 1589. 10.1038/s41467-021-21841-w 33707434 PMC7952727

[B167] Zhang Y. Zhang Z. Wang J. Zhang X. Zhao J. Bai N. (2022). Scutellarin alleviates cerebral ischemia/reperfusion by suppressing oxidative stress and inflammatory responses *via* MAPK/NF-κB pathways in rats. Environ. Toxicol. 37 (12), 2889–2896. 10.1002/tox.23645 36036213

[B168] Zhang J. J. Luo Q. S. Li Q. Q. Xu Q. Geng X. Xiong J. H. (2024). Fabrication and characterization of TPGS-modified chlorogenic acid liposomes and its bioavailability in rats. RSC Adv. 14 (35), 25289–25300. 10.1039/d4ra04116j 39139236 PMC11320192

[B169] Zhang C. Lan X. Wang Q. Zheng Y. Cheng J. Han J. (2025a). Decoding ischemic stroke: perspectives on the endoplasmic reticulum, mitochondria, and their crosstalk. Redox Biol. 82, 103622. 10.1016/j.redox.2025.103622 40188640 PMC12001122

[B170] Zhang L. K. Liu L. Li Z. Zhang Y. Zhai L. Zhang L. (2025b). Polyphenylalanine-baicalein nanomicelles reduce nerve cell apoptosis and inflammation to enhance neuroprotection and poststroke rehabilitation. Biomacromolecules 26 (2), 1149–1160. 10.1021/acs.biomac.4c01473 39874462

[B171] Zhang S. Li R. Song M. Han J. Fan X. (2025c). Exploration of M2 macrophage membrane as a biotherapeutic agent and strong synergistic therapeutic effects in ischemic stroke. J. Control. release official J. Control. Release Soc. 378, 476–489. 10.1016/j.jconrel.2024.11.033 39561947

[B172] Zhao L. Chen Z. Zhao Q. Wang D. Hu R. You Q. (2011). Developmental toxicity and genotoxicity studies of wogonin. Regul. Toxicol. Pharmacol. RTP 60 (2), 212–217. 10.1016/j.yrtph.2011.03.008 21459121

[B173] Zhao T. Tang H. Xie L. Zheng Y. Ma Z. Sun Q. (2019). Scutellaria baicalensis georgi. (lamiaceae): a review of its traditional uses, botany, phytochemistry, pharmacology and toxicology. J. Pharm. Pharmacol. 71 (9), 1353–1369. 10.1111/jphp.13129 31236960

[B174] Zhao Q. Lu F. Su Q. Liu Z. Xia X. Yan Z. (2020). Knockdown of long noncoding RNA XIST mitigates the apoptosis and inflammatory injury of microglia cells after spinal cord injury through miR-27a/Smurf1 axis. Neurosci. Lett. 715, 134649. 10.1016/j.neulet.2019.134649 31778769

[B175] Zhao K. Zhang J. Zhou L. Sun Z. (2024). Scutellaria baicalensis and its flavonoids in the treatment of digestive system tumors. Front. Pharmacol. 15, 1483785. 10.3389/fphar.2024.1483785 39654621 PMC11625591

[B176] Zhou X. Zhu L. Wang L. Guo B. Zhang G. Sun Y. (2015). Protective effect of edaravone in primary cerebellar granule neurons against iodoacetic acid-induced cell injury. Oxidative Med. Cell. Longev. 2015, 606981. 10.1155/2015/606981 26557222 PMC4628655

[B177] Zhou L. Han Y. Yang Q. Xin B. Chi M. Huo Y. (2022). Scutellarin attenuates doxorubicin-induced oxidative stress, DNA damage, mitochondrial dysfunction, apoptosis and autophagy in H9c2 cells, cardiac fibroblasts and HUVECs. Toxicol. vitro Int. J. Publ. Assoc. BIBRA 82, 105366. 10.1016/j.tiv.2022.105366 35470029

[B178] Zhu Z. Zhao L. Liu X. Chen J. Zhang H. Zhang G. (2010). Comparative pharmacokinetics of baicalin and wogonoside by liquid chromatography-mass spectrometry after oral administration of Xiaochaihu Tang and Radix scutellariae extract to rats. J. Chromatogr. B, Anal. Technol. Biomed. life Sci. 878 (24), 2184–2190. 10.1016/j.jchromb.2010.06.021 20643590

[B179] Zhu H. Guan J. Zhang H. Chang S. Wang L. Shi J. (2020). Simultaneous determination of ferulic acid, paeoniflorin, and albiflorin in rat plasma by ultra-high performance liquid chromatography with tandem mass spectrometry: application to a pharmacokinetic study of Danggui-Shaoyao-San. J. Sep. Sci. 43 (11), 2053–2060. 10.1002/jssc.201900846 32112520

[B180] Zong P. Feng J. Yue Z. Li Y. Wu G. Sun B. (2022). Functional coupling of TRPM2 and extrasynaptic NMDARs exacerbates excitotoxicity in ischemic brain injury. Neuron 110 (12), 1944–58.e8. 10.1016/j.neuron.2022.03.021 35421327 PMC9233078

[B181] Zou Y. Pei J. Wan C. Liu S. Hu B. Li Z. (2024). Mechanism of scutellarin inhibition of astrocyte activation to type A1 after ischemic stroke. J. stroke Cerebrovasc. Dis. official J. Natl. Stroke Assoc. 33 (3), 107534. 10.1016/j.jstrokecerebrovasdis.2023.107534 38219378

